# Barriers and facilitators for cardiopulmonary resuscitation discussions with people with heart failure

**DOI:** 10.1371/journal.pone.0314631

**Published:** 2024-12-31

**Authors:** Matilda M. M. Barnes-Harris, Sushma Datla, Alexandra Abel, Andrew L. Clark, Miriam J. Johnson

**Affiliations:** 1 Wolfson Palliative Care Research Centre, Hull York Medical School, University of Hull, Hull, United Kingdom; 2 Yorkshire and Humber Deanery, Leeds, United Kingdom; 3 Yorkshire and Humber General Practice Specialist Training Programme, Leeds, United Kingdom; 4 Hull University Teaching Hospitals NHS Trust, Hull, United Kingdom; 5 Wolfson Palliative Care Research Centre, Allam Medical Building, University of Hull, Hull, United Kingdom; University Medical Centre Ljubljana (UMCL) / Faculty of Medicine, University Ljubljana (FM,UL), SLOVENIA

## Abstract

**Background:**

Care planning with people with advanced heart failure enables appropriate care, and prevents futile interventions, such as cardio-pulmonary resuscitation (CPR).

**Aim:**

To explore what motivates clinicians to conduct, and people with heart failure and their carers, to engage in well-conducted CPR discussions.

**Methods:**

In-depth remote interviews with i) people with heart failure and self-reported daily symptoms (≥ 3 months), ii) informal carers and, iii) clinicians recruited through social media and professional groups, team contacts and snowballing. Interviews were audio-recorded, transcribed, anonymised and subjected to framework analysis. Findings were mapped to the Capabilities, Opportunities, Motivation-Behaviour change model.

**Results:**

Two themes were generated from 23 interviews: a) the cardio-pulmonary resuscitation discussion: preparation; who should conduct discussions; what should happen during discussions; impact on future discussions; b) Understanding of the: patient’s health status; and purpose and likely outcome of cardio-pulmonary resuscitation. For clinicians, ensuring preparation time, education, and support provided *physical and psychological capability*. For all, constructive experiences and a realistic understanding of health status and likely cardio-pulmonary resuscitation outcome *motivated* engagement in cardio-pulmonary resuscitation discussions providing *opportunity* for patient involvement in decision-making.

**Conclusions:**

For all, constructive past experiences of important conversations *motivates* engagement with CPR discussions. A realistic understanding of health status and likely cardio-pulmonary resuscitation outcome (all stakeholders), and training, skills, preparation and multidisciplinary support (clinicians) provide *physical and psychological capability*. Findings should inform organisational structures and training to ensure *opportunity* for this important clinical practice to take place.

## Introduction

Over 900,000 people live with heart failure in the UK [1]. Only around 13% survive for 10-years or longer [2], and many live with end-stage symptomatic disease for months or years. Care planning is important to ensure patients receive appropriate supportive and palliative care [3]. This prevents inappropriate cardio-pulmonary resuscitation (CPR) attempts and other invasive interventions [4]. However, clinician and patient understanding of disease trajectory, symptom burden, and CPR “success” is poor, with differing expectations about possible benefits [5–7], and challenges with communication and conflict resolution over decision-making, especially around end-of-life issues [8].

Resuscitation is rarely successful in patients with heart failure who have a cardiac arrest in hospital [9]. However, most people with heart failure believe that over half survive to discharge [7]. Patients with advanced heart failure given detailed information are more likely to opt out of CPR than those with less [10]. Clinicians’ understanding is also poor, with almost one third thinking a do not attempt CPR meant “no treatment”, including antibiotics, or hospital admission for any reason [11]. The American Heart Association recommends communication skills training for clinicians to support discussions about end-of-life care [6].

Studies and clinical guidelines recommend that advance planning, including CPR discussions, should be undertaken early [12, 13]. However, determining the best time for an individual can be difficult [14, 15]. The American Heart Association suggests that a routine annual heart failure review is the best setting, allowing discussions to be re-visited, rather than introduced for the first time during acute deteriorations [6]. However, time pressures and the sensitive nature of the discussions, means they are often not prioritised [6].

The Resuscitation Council UK has updated CPR guidelines to cover legal issues and provides a decision-making framework [16], but there are few published data regarding CPR decision-making in patients with advanced heart failure [17]. We aimed to explore the barriers and motivating factors to systematic and well-conducted timely CPR discussions in clinical practice for people with self-reported symptomatic heart failure, family carers and clinicians.

## Methodology and methods

We conducted in-depth telephone/video semi-structured interviews in a convenience sample of respondents to an online survey (survey not reported in this paper) of views and experiences of CPR discussions in clinical practice supplemented with purposively sampled participants recruited through team members’ networks. The interviews explored the barriers and facilitators to systematic, timely Do Not Attempt-CPR joint decision-making in clinical practice for people with self-reported symptomatic heart failure.

Inclusion criteria were: adults (18 years or older); members of the lay public with self-reported symptomatic heart failure (daily symptoms of heart failure over the past 3 months); and informal carers, family or friends and clinicians (medical and non-medical) caring for someone with symptomatic heart failure, and sufficient English to take part in an interview.

Participants from the team’s personal networks were sampled against the following characteristics; clinician specialty, seniority and profession, and carer.

### Data generation

The survey was promoted using the Qualtrics platform through social media (including Twitter, Facebook), professional bodies (the British Society for Heart Failure) and team members’ networks. Age, gender, and experience of heart failure were recorded. Participants interested in being interviewed to discuss their views and experience in depth could leave contact details at the end. Individual interviewing was chosen to allow open expression of views unaffected by others in a group where differential power dynamics between participants may impact freedom to speak.

Those willing to be interviewed were sent a participant information sheet and consent form and a time arranged at their convenience. Verbal consent was recorded prior to the interview. They were aware the interviewer was a clinical doctor and researcher, but not given any information about MB-H’s background. A topic guide was developed by the research team and piloted prior to use with a resident doctor; no changes were required in the topic guide and data from the pilot interview was included in the dataset. The Capability, Opportunity, Motivation–Behaviour framework informed the topic guide [18]. This model of behaviour proposes that a person will only engage in a specific behaviour (such as having a CPR discussion) if the person (person with heart failure, family member or clinician) has the capability (physical and psychological) and opportunity, and is motivated to engage in a certain behaviour (CPR discussion) more than another (e.g. delaying or avoiding a cardio-pulmonary resuscitation discussion) [18, 19]. New issues arising during interviews added to the topic guide for subsequent interviews.

Interviews were audio-recorded and verbatim transcribed using HYMS Microsoft Teams, and checked by MB-H (qualitative interviewing trained). Transcripts were anonymised by allotting a study ID and removing references to any identifiers. Participants did not receive transcripts for checking or provide feedback on the findings.

### Analysis

We estimated that a sample between 15 to 25 to provide sufficient information power and data saturation given the focussed topic [20]. Data were managed using NviVO 12 software.

The anonymous transcripts were subjected to the principles of framework analysis [21] using both inductive and deductive approaches, through the lens of Capability, Opportunity, Motivation–Behaviour. As part of data familiarisation, six transcripts were independently line-by-line coded by MB-H and one other researcher (SD, AA or MJ) and a code-book agreed. In an iterative fashion, codes were crafted together to form an initial framework of descriptive themes. M B-H then coded all transcripts using the codebook, applying the framework to all the data, whilst still allowing for new codes. Then, with discussion with MJ, key learnings from the earlier steps were generated, identifying key concepts helping to explain attitudes, experiences and behaviours were mapped to Capability, Opportunity, Motivation–Behaviour domains and further interpreted with the whole research team. This further discussion recognised that M B-H and MJJ brought their particular professional and personal experience to the analysis. Of note, M B-H conceived the project in response to her experience as a newly qualified doctor working in hospital cardiology care and witnessing absent, delayed or otherwise poorly conducted DNA-CPR discussions, often defaulting to herself due to her interest in palliative care. MJJ has decades of experience of championing integrated palliative care for people with advanced heart disease and is an advocate for excellent DNA-CPR discussions in response to witnessing the impact of poor practice. ALC is a senior cardiologist experienced in the daily practical challenges of prognosticating and planning in this patient group as well as knowledge of the training and skills expected for cardiology doctors. SD and AA are junior doctors training in cardiology, with less experience in palliative care.

### Ethics

Hull York Medical School ethics approval was in place prior to study start (REF 21–22.34). As recruitment did not involve health services, Health Research Authority NHS ethical approval was not required.

## Analysis report

Fifty survey respondents volunteered contact details indicating willingness to participate in an interview, of whom 13 responded to being sent the information sheet and consent form, and subsequently interviewed. A further 10 participants were recruited through the personal networks of the research team. Reasons for declining to participate were not sought.

All but one interview lasted between twenty and thirty minutes, and were conducted between 20^th^ April to 1^st^ July 2022 The first interview was a pilot, and only lasted ten minutes, however, as the topic guide did not require any changes, the data were included in the dataset even though it was short and the data relatively sparse.

The characteristics of the 23 interviewees are shown in Table 1. In order to balance the need to situate the participant group with the need for anonymity we have presented aggregate categories only. There was an almost equal gender representation and a broad range of clinician discipline and specialty. Some clinicians also had experience of caring for a family member with heart failure (these were counted in both categories, hence the total is greater than 23).

**Table 1:** Participant characteristics.

Interviews Demographics	Number (%)
Gender female	13 (57)
Age (years)	
20–29	1 (4)
30–39	5 (22)
40–49	8 (35)
50–59	4 (17)
≥60	5 (22)
Role	
Primary Care	3 (12)
Junior Doctor	3 (12)
Consultant	5 (20)
Nurse	7 (28)
Carer/family	3 (12)
Person with heart failure	4 (16)
Specialty	
Palliative	3 (20)
Cardiology	7 (47)
Other*	5 (22)

### Initial framework

Two descriptive themes were generated from the data; 1) cardio-pulmonary resuscitation discussion and 2) understanding. Theme 1 had four subthemes: preparation; who does it; what should happen; experiences. Theme 2 had two subthemes: person’s health status and understanding of cardio-pulmonary resuscitation. Themes, with subthemes and illustrative quotes are shown in S1 Table and are summarised in Table 2.

**Table 2:** Descriptive themes.

Theme	Summary description
**Theme 1. Cardio-pulmonary resuscitation discussion:** What happens before, during and after a cardio-pulmonary resuscitation discussion has impact on the benefits or harms that result. This theme relates to issues in relation to the cardio-pulmonary resuscitation discussion (before, during, outcomes).	
*Preparation*	• Discussions are more constructive when the clinician has considered issues in advance, including: cardio-pulmonary resuscitation -related ethics and law; the impact of privacy, venue and whether family can be present in person; the best timing, both time of day, and stage of disease. • Record taking must be clear and accurate, which may be supported by standard templates such as the Recommended Summary Plan for Emergency Care and Treatment (ReSPECT) form. • Clinicians need adequate time and space, access to resources such as information leaflets and be adequately trained and experienced.
*Who does it*?	• Consideration should be given as to which member(s) of the multidisciplinary team should be present, and who should lead the discussion on any particular occasion. • The level of seniority or speciality is less important.
*What should happen*?	• Rapport and trust should ideally be built in advance of the discussion, although not always possible. • Issues in relation to cardio-pulmonary resuscitation should be discussed as part of a treatment plan, over a number of clinical interactions, focussing on holistic patient care. • The patient and family should be involved, and care taken to discover their ideas, concerns and expectations. • If cardio-pulmonary resuscitation is considered to be futile, then the clinician should be clear that they are explaining a treatment option rather than asking permission. • Truthful and realistic expectations should be negotiated and common ground found.
*Experiences*	• The consequences of poorly conducted conversations can be serious. • Emotions of all stakeholders can prevent discussions due to fear, guilt, and not wanting to cause upset, compounded by society’s reluctance to talk about death. • The culture of all stakeholders can help or hinder cardio-pulmonary resuscitation discussions because of its effect on beliefs around death, the understanding of what cardio-pulmonary resuscitation is, and what it can achieve.
**Theme 2. Understanding.** A realistic understanding by clinicians, patients and families surrounding disease burden, prognosis, the purpose of cardio-pulmonary resuscitation and its success rates helps informed decision making and reduces the likelihood of harm.	
*Person’s health status*	• Symptom burden of advanced heart failure and comorbidities must be taken into account when assessing the likely outcome of cardio-pulmonary resuscitation. • How well a cardio-pulmonary resuscitation discussion is received depends on a joint understanding of the patients’ health. If these are not aligned the discussion can cause upset. If patients do not feel they are unwell enough to be discussing cardio-pulmonary resuscitation they are unlikely to engage. • Clinicians can find it difficult to recognise poor prognosis, especially if both patients and clinicians are inappropriately optimistic. • Cardiac devices can trigger cardio-pulmonary resuscitation conversations, but also can complicate cardio-pulmonary resuscitation discussions as specialist knowledge is required.
*Understanding of cardio-pulmonary resuscitation*	• The purpose of cardio-pulmonary resuscitation, and possible and most likely outcomes of cardio-pulmonary resuscitation in the individual–given their health status–needs to be understood and explained, and should be part of clinicians’ training. • Training in cardio-pulmonary resuscitation is universal, but specific communication skills in cardio-pulmonary resuscitation discussions should also be included. • Over-optimistic views of what cardio-pulmonary resuscitation may achieve is aggravated by media portrayals of rapid recovery, high success rates and poor technique. • The media, however, could be used to increase awareness of advanced decisions and appropriate understanding of cardio-pulmonary resuscitation outcomes

### Capability, opportunity, motivation–Behaviour mapping

The findings identified were mapped to Capability, Opportunity, Motivation–Behaviour model of behaviour (Table 3). This identifies the barriers and facilitators which impact the motivation and ability of clinicians, carers and those with heart failure to have a discussion about cardio-pulmonary resuscitation.

**Table 3:** Mapping of the themes to capability, opportunity, motivation–behaviour.

Capability, Opportunity, Motivation–Behaviour Mapping	
Physical capability	• clinician has been trained in the techniques, appropriateness of, and communication skills required for cardio-pulmonary resuscitation discussions and documentation • adequate knowledge and training of ethics and law around cardio-pulmonary resuscitation decisions.
Psychological capability	• the person understands correctly and accepts that their/the patient’s heart failure is progressing • the person understands correctly and accepts that a do not attempt cardio-pulmonary resuscitation decision is not “giving up”, or refer to all treatment and does not indicate lack of care or concern
Physical opportunity	• there is a private space available for the discussion to take place. • the clinician has adequate time to have the discussion. • resources available to support discussions (e.g. information leaflets, multidisciplinary team support) • IT support and clear, concise documentation are easily accessible • ReSPECT form or specific do not attempt cardio-pulmonary resuscitation form are used • data sharing capability within and between healthcare settings must be present
Social opportunity	• family involvement in discussions • media impact on cardio-pulmonary resuscitation understanding • impact of culture on beliefs around death and cardio-pulmonary resuscitation
Reflective motivation–cognitive responses in relation to cardio-pulmonary resuscitation discussions	• clinicians should evaluate health status and plan for current and future events in response to a person with heart failure’s understanding around cardio-pulmonary resuscitation. • recognising that open conversation can address fears without taking away hope. • recognise and respond to triggers that motivate someone to have a conversation about cardio-pulmonary resuscitation • Previous good experience improving motivation to have future cardio-pulmonary resuscitation discussions
Automatic motivation–Emotional responses in relation to cardio-pulmonary resuscitation discussions	• acknowledging emotional responses of people to difficult discussions. • fear of taking away hope or dealing with emotions can act a barrier to cardio-pulmonary resuscitation discussions • previous poor experience can act as a barrier to cardio-pulmonary resuscitation discussions.

**Fig 1:**
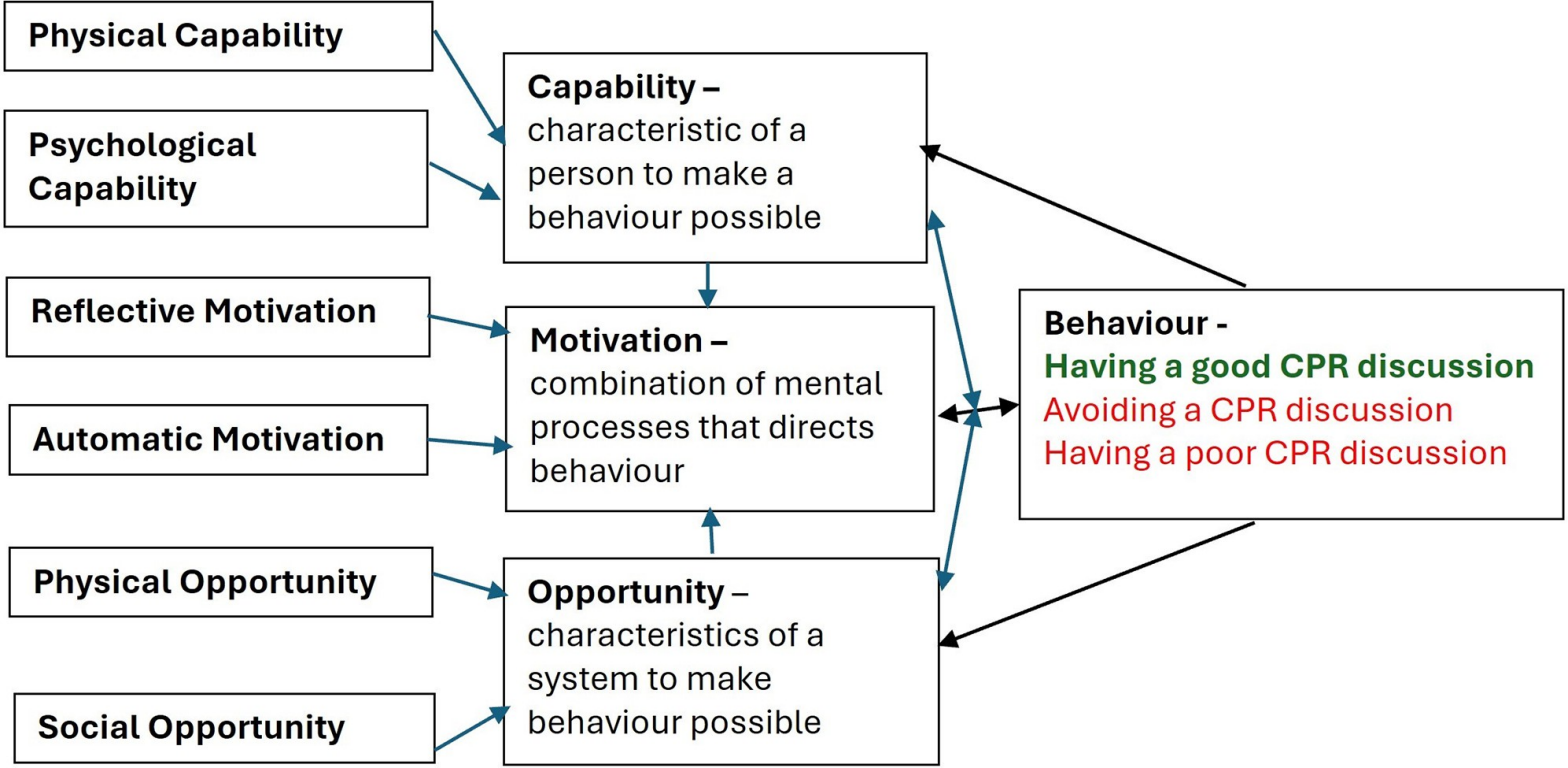
Capability, opportunity, motivation–behaviour model of change. A ‘good’ or ‘bad’ (in red) behaviour (cardio-pulmonary resuscitation discussion) is more likely to occur when someone has the capability and opportunity to engage and the motivation to undertake the discussion. Having adequate space, time, resources, social support, training will all encourage a good cardio-pulmonary resuscitation discussion, while not having this will increase the likelihood of avoiding discussions or having a poor-quality conversation. The discussion itself then feeds back onto someone’s motivation for future discussions and their awareness of their ability and requirements for these conversations.

This is summarised in Fig 1.

### Capability

To undertake a good cardio-pulmonary resuscitation discussion the clinician must prepare thoroughly, ensuring they understand the ethics and law surrounding withholding of treatment, consent and the relevant documentation (e.g. ReSPECT form. Advanced Directives to Refuse Treatment). This can be challenging, especially in cases where capacity isn’t clear. It is also helpful to identify any misunderstandings the patient or family may have with this regard, for example, believing that a DNA CPR decision equates to ‘no further care’ or even assisted dying.

*‘Well, it depends on if there’s a proxy decision maker that’s in place or suppose if there’s a lasting power of attorney, they are technically the patient in terms of decision making and so it would be them if that was official and registered.’* Palliative consultant [ID2]

All interviewees agreed that all clinicians should be able to talk about cardio-pulmonary resuscitation, supported by the multidisciplinary team. A sufficiently trained, confident individual with good communication skills should lead the conversation. These skills were not always associated with seniority or specialty, and was enhanced by attentive preparation prior to the conversation, ensuring accurate and current clinical knowledge of the patient.

*‘I think people find that really difficult to talk about because, you know, we’re scientists at the end of the day. And I think having a conversation about what we think might happen in the future is more the art of medicine and seeing the progression of someone over time and especially for non-consultant grade doctors who potentially might meet this patient once you don’t have the benefit of a longitudinal view in the same way that an old-fashioned General [Family]Practitioner might have done. And so, I think those things make it really difficult to take the bull by the horns and say I’m going to do this because it’s the right thing to do for the patient.’* Cardiology registrar [ID5]

However, many reported that the discussions were happening too late, especially in clinical cultures where the emphasis is on finding another disease-modifying treatment to prolong life. Delaying conversations until they become unavoidable are not helpful:

*‘I don’t think a lot of cardiologists are very good at having these discussions, and that’s probably something about the fact that we’re quite an active specialty in that there are always things that we can do. And it’s my experience that quite often these discussions happen quite late, someone’s 80 and has got cardiogenic shock and they’re on inotropes before we think about having those discussions.’* Consultant cardiologist [ID13]

Implanted defibrillators were seen as a barrier to cardio-pulmonary resuscitation discussion, as specialist knowledge is required. However, those with this knowledge often used devices as a ‘way in’ to discuss cardio-pulmonary resuscitation status as part of a treatment plan.

Overall understanding of how heart failure might impact cardio-pulmonary resuscitation success was key for a good discussion:

*‘You’ve got to insist on shared understanding before you can reach decisions’* GP [ID14]

Conversely, a lack of a shared understanding was reported as a huge barrier to patients and family engaging in cardio-pulmonary resuscitation discussions. Cardiology team members described how they had known their patients for years. This built strong relationships, but this, paradoxically, made it challenging to talk about death and dying, with clinicians feeling they have failed when they finally discuss it.

*‘If you’ve been that person for that patient, for a number of years and you’ve always been the one who’s supported them or got on tablets and you’ve explained it; well we can try and do this, we can try and do that. I think sometimes there’s a fear that there’s almost a finality to it. Like, I’m sorry there’s nothing else I can do, we need to talk about CPR [cardio-pulmonary resuscitation] now, and that’s not necessarily the case. But it does make you push it that little bit further on down the line until you really have to do it and that’s possibly not a failing, I think that’s a human thing because you don’t want to give people bad news. But at the same time, because you’ve built up sort of four years of a relationship to then suddenly say this is it.’* Heart failure nurse [ID11]

Some clinicians avoided the conversation completely with detrimental outcomes. A mutual unspoken ‘pact’ between patient and clinician to maintain a positive outlook was reported, leading to poor outcomes for the patient:

*‘I think I’ve probably seen episodes over the years, which probably don’t align with best practice where clinicians have kind of colluded with patients and agreed that they could still stay for CPR [cardio-pulmonary resuscitation] even if it wasn’t appropriate and is unlikely to be successful. And then anecdotally, colleagues have seen that as well where patients have been subjected to CPR after they’ve died, where the likelihood of success is close to zero. And people kind of go through two or three cycles of CPR almost for the sake of it and then step away and call the patient’s death at that stage. And which you know when you hear about it and you think about what’s involved in doing that process, it’s quite inhumane to think that that’s going on or has been going on.’* GP [ID13]

### Opportunity

Prior to a cardio-pulmonary resuscitation discussion, clinicians must consider the best timing of the conversation including whether family members can be present. During COVID many patients were unable to have family present with adverse effects on discussions.

*‘during COVID it was difficult for those conversations to be initiated”* Heart failure nurse [ID13]

*‘we couldn’t have their relatives there and it was a complete disaster*.*’* Cardio cons [ID5]

However, family could also be a barrier, emphasising how conversations need to be tailored to the individual.

*‘Although sometimes that’s [having relatives present] not actually helpful because occasionally patients feel a little bit more free*, *I think to talk about dying without relatives there*.*’* Cardiology doctor [ID10]

However, most often family presence was valued by clinicians, family and those with heart failure, and helped family understand the clinical situation better.

*“we must identify that this person is deteriorating early, allow the family to be at the bedside and that the medical team are present to talk through why these stages [of heart failure] cannot be undone so that they can see it’s not that people have given up”* Cardiology registrar [ID5]

Poor documentation and communication of the cardio-pulmonary resuscitation status was raised. Without universal access to clearly documented forms, clinicians revisited decisions, undermining patients’ trust in the clinicians.

*‘…information sharing to improve across provider organizations, one record will be lovely. And coding of information as we complete an advanced care plan, there are coding opportunities around resuscitation and escalation of care. Special notes can be added into system one, so paramedics can see that and other healthcare practitioners. The sharing of information with care homes in particular on discharge from hospital and moving away from paper-based respect forms which just seems so archaic, to electronic forms that can be seen across the system.’ Care of the elderly* Consultant [ID22]

The media was reported as creating false optimism and seen as a large barrier. Most TV soaps show poor cardio-pulmonary resuscitation technique and a rapid recovery back to–often–a better quality of life than the patient had prior to arrest. Respondents felt this not only gave unrealistic expectations, but also missed an opportunity for effective society education particularly around the purpose, extent and scope, and effectiveness of appropriate healthcare.

*‘I think people seem to have false perceptions from TV and film, of what we can actually be achieved and what it’s for.’* Emergency Medicine Consultant [ID3]*‘…is CPR [cardio-pulmonary resuscitation] the symptom of a bigger problem that we need to be talking to people about? What is the point, the purpose of healthcare, what we need is to renegotiate expectations with individual patients but also with society about what the health service is there to do and not to do.’* GP [ID16]

Having these conversations was seen as part of a broader discusion around treatment. Clinicians need to plan for current and future events, ensuring patients and carers fully understand, starting earlier in the disease trajectory.

*‘a drip feed, that seed could be sewn quite early on’.* [ID4]

### Motivation

A cardio-pulmonary resuscitation discussion should be between a clinician the patient trusts and the person with heart failure, preferably, albeit not always possible, over multiple interactions.

*‘So there’s this element of trust, I’ve entrusted these people to act in the best interests of my relative. And I would hope that they are going to take the correct decision at the time, which is based on not just the purely the medical outcome, that it’s based on the quality of life, type of decision.’* Carer [ID8]

Clinicians found the most challenging discussions often came from asking patients whether they would like cardio-pulmonary resuscitation when it was inappropriate. If the conversation was phrased as a request for a patient’s wishes, rather than placed in the context of what treatment was appropriate and would be offered in the context of advanced disease, then this led to misunderstandings and difficult conversations.

*‘I think there’s also an awful lot about the way the question is phrased and how you bring it up. So, talking about resuscitation as though it is a good treatment and an option for patients actually is often the way of dealing with things and sometimes framing it as a medical decision for a treatment that you think is futile for a patient that is dying is a better way of bringing that up.’* Cardiology consultant [ID10]

Many people reported uncertain prognosis as a barrier for cardio-pulmonary resuscitation discussions.

‘*I think acknowledging uncertainty with patients is important, but can also be quite anxiety provoking, you know, like I think if you said to each patient what you thought their likely prognosis is, firstly you’re probably wrong. But secondly, it’s not, going to be kind of positive interaction really for that patient, unless you can say something a bit more concrete and for that you need a trajectory’.* Cardiology registrar [ID9]

Clinicians’ fear of upsetting patients or family, patients’ fear of death, and guilt about giving up and fear of the unknown were major obstacles. Underpinning the emotion was often a poor understanding of cardio-pulmonary resuscitation and health status, a lack of preparation and privacy. Conversations in a ward of people all with similar conditions, behind thin curtains so all can hear ‘upsets the whole ward’, not just the individual. Conversely, realistic understanding enabled a more positive experience. Emotion shouldn’t stop these conversations from happening, and in fact many reported they felt relief, and anxiety can be eased by giving clear information to people with heart failure in an appropriate setting.

*‘I think you have to be very sensitive and compassionate and understand that it can be a very upsetting and distressing conversation, but equally it can be a very calm and considered conversation where actually that person already knows that they wouldn’t want that procedure to happen.’* Palliative care nurse [ID21]

## Discussion

We found that a well-conducted and constructive cardio-pulmonary resuscitation discussion is facilitated by appropriate clinician training and skills, including communication skills, rather than seniority or specialty alone. An understanding of the legal and ethical aspects and of the likely outcome in an individual given their health status is a key preparatory step. The conversation should be part of ongoing management discussions, preferably with a trusted clinician, in a suitable environment, and supported by the MDT. Patients and families with a realistic understanding of the individual’s health status and the likely outcome of a cardio-pulmonary resuscitation attempt, are more likely to engage usefully with such discussions. These physical and psychological “capabilities” motivate good practice and foster a joint understanding and way forward, and a helpful experience. This in turn acts as motivation for future “good behaviours”. However, the converse is seen. Poor preparation, training, skills and rapport with a poor understanding of the likely success from a cardio-pulmonary resuscitation attempt by clinician, patient, family or all three, lead to destructive experiences for all, feeding a sense of betrayal and confusion (patient, family), failure, guilt and helplessness (clinician), and distress (all). This de-motivates, leading to delay or avoidance of, or poor engagement with, discussions, with potentially serious consequences for the patient and family.

There is a significant literature identifying the necessary components of ‘difficult’ conversations [22] or ‘serious illness’ conversations [23] and showing the value of communication training interventions [24]. Our findings regarding what makes a constructive cardio-pulmonary resuscitation conversation are consistent with previous reports. Hospice nurses, who have day-to-day experience of patients with very advanced disease and for whom advance planning conversations are a core component of their work, are more confident than heart failure specialist nurses in end-of-life discussions [25]. Nurse-led discussions, focused on patient autonomy, are more likely to result in patients wanting cardio-pulmonary resuscitation compared to physician-led discussions focused on patient harm-benefit balance [26]. Studies in patients with other chronic conditions, such as multiple sclerosis, also emphasise the importance of appropriately trained professionals leading cardio-pulmonary resuscitation discussions as part of continuing care [27].

However, there are fewer data regarding the effective implementation in practice, although the clinical culture is highlighted as a particular challenge overcome [28]. Our demonstration of the positive and negative “feedback” loop consequences of “good” and “bad” conversations on clinician and patient/family conversation related behaviour is novel. In particular, we show the key impact of the first conversation a clinician witnesses or leads and the importance of whether such conversations are given priority within a clinical culture. If the culture is one where care and time are taken, ‘good’ conversations are modelled by seniors, who also support their junior staff to do this well, then this acts as a virtuous cycle, motivating the more junior clinician to take time and care, and conduct ‘good’ conversations. This is a fundamental and pivotal point of implementation, that goes beyond communication skills training and would help to prevent a vicious cycle of avoidance for fear of conflict.

Consistent with other studies we found that where, when and by whom cardio-pulmonary resuscitation conversations take place is patient specific [29]. If possible, conversations should be outside the context of an acute illness, in a calm and quiet environment with a trusted clinician with good communication skills [1, 30, 31]. Advance care planning conversations, including cardio-pulmonary resuscitation, part of ongoing heart failure management provided by a multidisciplinary team with integrated palliative care appear to be optimal [4, 32] Honest conversations about the stage of disease and the limits of clinical medicine may also help assuage the moral distress we identified in our data. Moral distress is common, not only in situations where clinicians feel the patient did not receive the interventions they should have, but also in situations where they felt futile, but burdensome interventions were misused and misapplied [33]. Clear decision-making ‘in hours’ should help prevent moral distress for clinicians (often junior, often with less knowledge of the patient) faced with acute clinical deterioration ‘out of hours’ [34]. Such decision-making is complicated by a commonly poor understanding of the ethics and law surrounding the decision, both by patient and family, but also by clinical staff. Fear of legal censure, and precipitating complaints, may lead to avoidance of such conversations, risking the further ethical challenge of then allowing a patient to receive futile and potentially harmful interventions [35].

The primary care doctor is well-placed to initiate honest conversations as a trusted clinician with long-term knowledge of the patient in the context of their family [36]. However, this may be undermined by poor information sharing or access about disease stage, or treatment plans. Good technology systems would support clinicians’ access to key and timely information [37, 38].

We found patients’ and carers’ understanding of their health status influences how cardio-pulmonary resuscitation is viewed, consistent with work showing that people with multiple conditions were more accepting of do not attempt cardio-pulmonary resuscitation decisions [26]. Cardio-pulmonary resuscitation was initially intended for people experiencing cardiac arrest due to drowning or other reversible acute insults [39]. Outside of that context, it commonly causes significant morbidity in survivors [40]. False optimism regarding both restoration of sinus rhythm and quality of life is reported in many chronic conditions; collusion between clinicians and patients with mutual denial of likely poor outcomes is common [41, 42]. We found that the media was identified as a source of erroneous information, and raised expectations consistent with previous reports [43], but it could be used positively to create opportunity by educating society about a more accurate cardio-pulmonary resuscitation success rate, good technique and the likely post- cardio-pulmonary resuscitation intensive care required [44]. An example of how media can be used to promote accurate and beneficial health messages was seen during the COVID pandemic [45].

We found that clinicians, people with heart failure and family thought involving family was important, but the interplay between patient, family and clinician can be complex. A systematic review of 20 papers about cardio-pulmonary resuscitation discussions with people with a range of serious illnesses found that involvement of family members was seen as positive by the overwhelming majority [29]. However, many people with advanced disease wish to avoid burdening family with decision-making during an acute deterioration [46]. Advance care planning was seen as a way to involve the family while the patient has capacity to express their future preferences [47–49]. Some people preferred their children to make medical decisions for them [46]. However, as was alluded to in our findings, difficulties arise when there are conflicts of interest or disagreements between family members and/or the patient [47–49].

### Strengths and limitations

We present findings from a range of clinicians, and people with personal experience of heart failure. Applying the Capability, Opportunity, Motivation–Behaviour lens brings a depth to our analysis, going beyond describing barriers and facilitators, to an understanding of what leads to behavioural change.

Recruitment through social media will have biased selection to those with sufficient technology skills, and resources; to those who have an interest in the topic; and those with greater self-efficacy. This is reflected in younger, healthier subjects in our study than most people with heart failure. Although we collected only minimal demographic data, our sample may also therefore be biased regarding education levels. Given the relationship between socio-economic status, education, the presence of chronic medical conditions and health literacy, our participants with personal experience of heart failure are more likely to be those with greater levels of self-management and advocacy. However, despite this, our findings regarding barriers and facilitators are consistent with other published literature. The research team members were all female clinicians, except for ALC (three junior doctors, a clinical professor in cardiology and professor of palliative medicine and mixed-methods academic), although the presence of both cardiology and palliative care viewpoints provides balance.

Our sampling strategy included both convenience and purposive approaches, the latter using the team’s personal networks. This may have introduced bias due to personal connection (at least indirectly, with ‘like-minded’ bias) with the research team.

### Implications for clinical practice and future research

If people with heart failure or clinicians do not have the capability or opportunity to have a cardio-pulmonary resuscitation discussion, neither will be motivated to initiate or engage with a conversation. Every experience of cardio-pulmonary resuscitation discussions will have an impact–good or bad–on future behaviour. Lack of opportunity might result in patients being unable to express their own wishes (conversation does not happen); opportunity coupled with poor capability (conversation happens but is done poorly) may result either in serious distress and broken relationships between clinicians and both patients and their carers; or in false optimism.

All clinicians should be trained and supported to undertake cardio-pulmonary resuscitation discussions with appropriate supervision when starting to learn this skill to ensure positive first experiences. Those with heart failure and their families need a good understanding of the impact the severity of their disease on the likely outcome of cardio-pulmonary resuscitation. They must be supported to ask questions when unsure and to take an active role in the management of their care.

Our findings may also be applicable to cardio-pulmonary resuscitation discussions with people with other diagnoses to improve engagement with these challenging conversations.

Future research should work with a representative sample of stakeholders, and further explore the challenge of embedding cardio-pulmonary resuscitation conversations in standard care.

## Conclusions

Constructive past experiences of important conversations *motivates* initiation or and engagement with cardio-pulmonary resuscitation discussions. A realistic understanding of patients’ health status and likely cardio-pulmonary resuscitation outcome (all stakeholders), and appropriate training, skills, preparation and multidisciplinary support (clinicians) provide *physical and psychological capability* for well-conducted conversations. These findings should inform health service structures and training to ensure the *opportunity* for this important part of clinical practice to take place.

## Supporting information

S1 File Topic guide. (DOCX)

S1 Table Themes, subthemes and illustrative quotes. (DOCX)
