# Supplementary material for: Barriers and facilitators for cardiopulmonary resuscitation discussions with people with heart failure
Source: PLoS One. 2024 Dec 31;19(12):e0314631. doi: 10.1371/journal.pone.0314631 (PMC11687877; doi:10.1371/journal.pone.0314631)
Supplement: S1 File — (DOCX) [file pone.0314631.s001.docx]

**Supplemental Information 1. Interview topic guide**

**Introduce researcher**

Check if any questions about the interview and record verbal consent. The process of verbal consent is:

1. Check happy to record and inform that recording has started
2. Reading the consent script and requesting verbal consent to each item

**Consent script**

1. Do you confirm that you have read the information sheet dated 07.04.2022 (version 1.0) for the above study?
2. Do you confirm you have had the opportunity to consider the information, ask questions and have had these answered satisfactorily?
3. Do you understand that your participation is voluntary and that you are free to withdraw at any time without giving any reason, without your legal rights being affected?
4. Do you agree that if the interview is terminated prematurely, that the data already collected will be used in the analysis?
5. Do you agree that anonymous quotations from this interview can be used in presentations or publications arising from this project?
6. Do you agree that anonymised data can be used by authorised researchers working on similar studies?
7. Do you agree to take part in the study?

**Interview topic guide**

1. Tell me about your experience of heart failure.
2. Tell me about what you understand about cardiopulmonary resuscitation (CPR) and what it is intended to achieve?
3. Probe – what is a “good”/”bad” outcome (e.g., brain damage, function, cognition, ITU)
4. Probe – where have you learned about CPR? (media [to those with heart failure/carers – but also HCPs], technical “how-to” mandatory training [CPR], other sources that might influence nuances about appropriateness [MDT discussions, pall care team, experience etc.], any good resources you found helpful).
5. Probe – what do you think the likely success is and why? (who would “come back to previous QoL” and who would not)
6. Tell me what you think about Do Not Attempt CPR discussions
   1. Probe – what makes a “good”/”bad” one?
   2. Probe – what skills do healthcare professionals need to do this well?
   3. Probe – at what point in the illness should these discussions happen (e.g., acute crisis, worsening symptoms, difficulties in managing treatment, recurrent admission)
   4. Probe – what are the best circumstances to have such conversations? (time, trusted HCP, invited by HCP or raise topic by those with heart failure)
   5. Probe – what are the consequences if i) not done or ii) done badly?
7. Tell me, if you are happy to, about any personal discussions you have had about CPR
   1. Probe – who with? (HCP, family, friends)
   2. Probe – under what circumstances?
   3. Probe – “good”/”bad” and what made it so?
   4. Probe - What benefits and burdens were discussed?
8. How do/should opinions of family members influence your decision regarding CPR?
9. If you (or those with heart failure you are caring for) were too sick to make a decision about CPR who would you like to make the decision for you and why?
10. Probe – family or clinician or independent advocate?
11. Any other thoughts, or anything you would like to talk about?

Thank you for taking part in this interview. I will now turn off the recording.

| Table. COM-B Mapping | |
| --- | --- |
| Physical capability | - 2b training regarding CPR prompt - 3b skills required for CPR discussion |
| Psychological capability | - 1 experience of heart failure - 2c likely success of CPR |
| Reflective motivation | - 3c triggers for conversation. - 4 personal experiences |
| Automatic motivation | - 3a what makes a good/bad conversation - 3e Consequences - 7 any other information – often brought up emotions. |
| Physical opportunity | - 3a what makes a good/bad discussion - 3d best circumstances for conversation - 4a personal experience |
| Social opportunity | - 2b learning for CPR eg media - 4a who have you had discussion with - 5 should family be involved - 6 who would you want to represent you/a patient if they didn’t have capacity. |
