# Supplementary material for: Barriers and facilitators for cardiopulmonary resuscitation discussions with people with heart failure
Source: PLoS One. 2024 Dec 31;19(12):e0314631. doi: 10.1371/journal.pone.0314631 (PMC11687877; doi:10.1371/journal.pone.0314631)
Supplement: S1 Table — (DOCX) [file pone.0314631.s002.docx]

**Supplemental Table. Themes, subthemes and illustrative quotes**

| **CPR DISCUSSION** | **Factors affecting the CPR discussion** | | **QUOTES** |
| --- | --- | --- | --- |
| **THEME 1 Preparation** | Ethics, Law | Knowledge of the law surrounding CPR decisions, capacity and when these discussions should be taking place and the ethics around not having these discussions appropriately | Ethically, the patient herself should be the decider and the family have an ambivalent role around that and it becomes very difficult once you move into other cultures. *General Practitioner*  We were trying to find out first of all, what does the patient know? What is their perception? And then we need to, from the ethical point of view, we need to establish how much do they want to know how much capacity have they gone to take on what you what they could be told. *Stroke Nurse*  And I think I’ve probably seen episodes over the years, which probably don't align with best practice where clinicians have kind of colluded with patients and agreed that they could still stay for CPR even if it wasn't appropriate and is unlikely to be successful. And then anecdotally, colleagues have seen that as well where patients have been subjected to CPR after they've died, where the likelihood of success is close to zero. And people kind of go through two or three cycles of CPR almost for the sake of it and then step away and call the patient's death at that stage. And which you know when you hear about it and you think about kind of you know, what's involved in doing that process, it's quite inhumane to think that that's going on or has been going on. *General Practitioner*  so not having at all, you're robbing somebody other opportunity to be able to express their own wishes, because by the time they need, it's often too late. *Emergency Department Consultant Physician*  So you were then left with trying to manage a patient who was dying, which in some ways I suppose gave the family a bit more time at times to say goodbye, but really is not in the patient's best interests. And. And yeah, it's brutal and being involved in the actual process of really horrible. I wouldn't want families to see it and I wouldn't want patients to watch it back because it's horrible. *Palliative Care Consultant Physician*  Unless you felt that they didn't have the best interest of the patient at heart, in which case you'd have to escalate that. But so it would be them. If that isn't in place and it would be a best interests decision and usually led by the treating medical team involving the family and all those closest to the patient. I guess if there wasn't anything anybody like that for the patient's whether other healthcare professional know them and whether you'd have to involve an IMCA [Independent Mental Capacity Advocate]. *Palliative Care Consultant Physician*  So, the discussion is about priorities of care and values and goals for care and if it's appropriate to, CPR may come into that that discussion. And then, so that becomes about priorities and choices and then, and obviously then the potential to record that conversation in a way that it hopefully informs future care. *General Practitioner*  So he signed the form, we signed the form, it got sent to everybody. I can't even remember the situation where the ambulance came, but there was a situation where he had night sitters, then he was so end of life care. … Somehow an ambulance got there. There was a signed form in the room and still the ambulance staff started CPR… So the good outcome is that the patients voice always gets heard and followed by everybody else which is why documentation in theory should be helpful. It's just in my experience which is I realize that's a one off, it doesn't always get followed. *General Practitioner*  And so she no longer had capacity and it was her two daughters I was having the conversation with. And one felt very strongly that CPR was not appropriate for her mum and the other felt very strongly that they were giving up on her mum by signing off as a Do not resuscitate and so the entire conversation in that case was with family because the woman concerned had no capacity at all… But yes, you know obviously a very complex and challenging decision for the family and also for the woman was in a care home, so the care home and clinical team involved. *General Practitioner*  I mean obviously at the end of the day, if I'm *compos mentis* and my wife is *compos mentis* and there's either my decision ideally or our decision and in that sense, the children are not involved because it's not their life, obviously, but they've got an emotional kind of purchase on it. *Patient*  I think that's part of the good thing is that we've got the lasting power of attorney and he's very clearly happy for me to make those kind of decisions. *Carer (oncology nurse)*  He'd had a treatment escalation plan put in place the family had objected to it. It had then been rescinded. And the patient was end of life. And it was really, really difficult. There was lots of hard feeling against the consultant who had rescinded the decision, having met the patient’s relative. I would have made the same decision and done exactly what he had done, which was at we must identify that this gentleman is deteriorating early, allow the family to be at the bedside and that the medical team are present to talk through why these stages cannot be undone so that they can see it's not that people have given up, it's just that there's not anything extra to do. *Cardiology Registrar Physician*  Well, well, it depends on if there's a proxy decision maker that's in place or suppose if there's a lasting power of attorney, they are technically the patient in terms of decision making and so it would be them if that was official and registered. *Palliative Care Consultant Physician* |
|  | COVID, privacy, venue | The impact COVID-19 has had on CPR discussions including where the discussions are held. | that definitely adds to complexity to those decisions if people come with the idea that we're just mean old nasty doctors who, … we just wanted to not treat people, I think it's probably been polarized by COVID and the decisions that were made early on in the pandemic on the basis of trying to protect the NHS, I think that's probably had an impact on our desire to make these decisions quickly when patients come in because there is a fear that we get accused of not doing as much as we should do. *Cardiology Consultant Physician*  So I think sometimes decisions at the moment are, will certainly in COVID, certainly seemed to have been made quite quickly and not necessarily wrongly, but the conversations maybe haven't been that supportive with patients maybe and they have come out of hospital with those decisions being made quite an acute phase that they've not really been involved in the communication process because I think that's crucial, that most of these patients understands why decisions are made. *Heart Failure Nurse*  And the one thing that due to COVID and primary care, the impact on primary care during COVID that although their GP has had phone calls with her had not eyeballed at, did not truly hadn't think appreciate the position she was in so we had a very frank and open conversation around what she would want and my professional opinion on where we were up to with this and if she wants to have cardiac arrest, what the outcome potentially would be and you know I was I was very honest with her and her family were there and they were all very much in agreement and she was quite adamant that she didn't want to go to hospital. *Heart Failure Nurse*  And but that sort of sticks in my mind because during COVID, it was difficult for those conversations to be initiated, by other primary care teams. So a lot of it did fall to us to maybe start those conversations and when you're quite close to someone that can be quite hard as well, because I've known this lady for a long time. *Heart Failure Nurse*  Particularly in the last say two years where even visiting is very difficult for us. *Heart Failure Nurse*  I think due to the pandemic, I think a lot of that has become much harder to do and might have been easier in a hospital setting. But I don't think, my parents would have struggled with that, with being in a hospital setting, I think they're probably preferred to be at home, *Carer*  I think there are lots of good things that have come from virtual working as a result of the pandemic. I think what has made it extremely difficult to do when you're on the other end of the phone and you know what you should be talking about, but you can't. And then when we were able to bring patients up to see us face to face, we couldn't have their relatives there and it was a complete disaster. *Cardiology Registrar Physician*  I actually prefer doing it with patients in clinic rooms rather than when they're very unwell because I think it feels quite, I think the patients can feel quite vulnerable when they're in hospital beds when you having those conversations. *Cardiology Consultant Physician*  I think ideally is probably in their own homes where they feel comfortable and there's no time constraints. There's no risk of fire alarms going off and there's no, you know. Ideally it's in an ideal world that isn't it, *Heart Failure Nurse*  you need to be in the right surroundings and it's a conversation that needs to be addressed, you know, because a lot of our patients, they're elderly, they can be hard of hearing and then you can have patients of different backgrounds, maybe English isn't their first language. So you know, and they may not, you know, it's important that maybe someone's there, that a second pair of ears that hears, you know that so that they when they go home, they can discuss it with them as well *Heart Failure Nurse*  The doctor won't take you out of the ward, and nowadays the doctor won’t even pull the curtains to ask you*. Patient*  But I think I should do it privately, not in front of everybody, cause it really upset me, I started crying when I heard, when they turned around and did it. And somebody told me, one of the nurses said that’s normal thing. They just ask you there and then in front of people. *Patient*  But I think wherever the patient feels most comfortable and essentially it's a difficult conversation about their future care and it should be had in the setting that they feel happy with. And for most people that's, you know, that's probably in a clinic room where whether that's a GP surgery or hospital outpatient clinic for a lot of people, it happens in hospital wards, doesn't it and A&E departments, where it's less than ideal. But yeah, in an ideal world, if the patient wants to have it in a clinic room, that's fine. I suppose most people would probably have it in their living room, but that's not quite feasible. *General Practitioner*  So yeah, you mentioned that the setting we have here, but a more relaxed I don't want to say healing environment, but certainly one without beepers and buzzers aren’t going off and interruptions and curtains and people on commodes and everything else. So it's somewhere like here like in a Health Center or GP practice or an outpatient setting is where it should be held. *Care of the Elderly Physician*  That's the lovely thing about here is that the relative is here, the wife of our daughter. It's more protected and supported. But also they feel. You know the rapports there, isn't it? It's quiet. There's no noise. *Heart Failure Nurse*  …ideally if they've got someone with them that that cares for them and that they can have kind of ongoing discussions with afterwards and if it's on the ward, that's a bit more challenging, because there often isn't anywhere apart from the bed space, but I think you can kind of make that environment a bit more, a bit more appropriate. You know, always making sure the curtains are drawn, you're the patient’s level, things like that and it's not ideal, but it's probably the best you can do. *Cardiology Registrar Physician*  …my strong feelings are around the advocacy side of things, I feel like if you don't have conversations when the patient is relatively well, then it leads to a conversation me doing a conversation in ED is suboptimal… when somebody is quite unwell. That's, any hospitals setting, the patient as an inpatient, even on a ward. You're still not able to give the time that you would want to. And give that kind of opportunity for them to give their own views and advocate for. You certainly can do a good imitation of that, or attempt at it, but it’s still suboptimal if you’re doing it in an inpatient setting like ED or a ward. *Emergency Department Physician*  But because my parents couldn't get to clinic, couldn't get to the surgery, it was a home visit and therefore it was just whenever the doctor could make the time and I'd stayed one day all day. I left about 4:45 on the evening and he turned up at 5:30. *Carer* |
|  | Timing | When a CPR discussion is had during the day, within a patient's trajectory. | And as I suppose, there's multiple different occasions when you might choose to talk to someone about their resuscitation status and what their wishes are. *Cardiology Registrar Physician*  I've seen so many patients with heart failure. Where you know, they have really bad quality of life they have. The they're struggling with sort of peripheral oedema. You get when you try to put in a cannula you can see that the fluid is oozing out and they're struggling with their breathing. And it's just not a good quality of life and I think. And they still they don't have DNA CPR in place. And I think I think discussions need to be had a lot before someone gets to that point … And when someone's on multiple agents in terms of medication, I think discussions need to be had. *Junior Doctor*  I guess as a general rule, I would really hope that patients with heart failure are aware that it's a life limiting diagnosis and whilst I know there are new things being done at all the time and new devices and things that can help prolong, prolong life, etcetera. And it's still will probably be their cause of death in the end and shorten their life. So I'd really hope from the outset almost that they're aware of that. And then I suppose as things progress, it's then deciding, I guess when that resuscitation discussion has to happen, but you'd hope you'd be doing it alongside other ACP discussions along the way. …It is hard to have specific triggers, but I guess potentially whether you'd think about, you know, someone who's had multiple hospital admissions or and someone who has progressed to third line drug treatments, that kind of thing would be a trigger to say right, would this patient be offered resuscitation if they arrested right now and have that conversation at that point? *Palliative Care Consultant Physician*  for some it is a, it is a shock and something very difficult to get their head around. Yeah, yeah, I guess seizing any opportunity you can along the way is helpful, isn't it? *Palliative Care Consultant Physician*  Sometimes that conversation has been initiated by patients. Sometimes it's been initiated by me, if I can see people getting worse over a number of months, that's obviously much easier if I know those patients. So having time. Having some space. Having patients who are engaged with that conversation almost before we start having it makes a really good conversation. *Cardiology Consultant Physician*  In hospitals, again, I suppose it's the same things if patients have come in with clear advanced heart failure and their prognosis is poor, we'll try and initiate that discussion. That can be quite difficult on a ward round if it's the first time you've seen someone and you've got twenty other patients to see. And when people are deteriorating on a day by day basis as well, that would be a time that I would try and bring it up. *Cardiology Consultant Physician*  Because if you give it too early, I think you can ruin what time they have because they said they think they're going to die when actually you know it is a very important conversation to have. But when you have it is the issue. When the patient doesn't feel ambushed by suddenly having someone come up and say I don't think we should perform if this happens because again, it's out of the blue. *Heart Failure Nurse*  The timing for me, I I would never discuss it right at the start. … There's things that we can actively put in place to try and improve function. So I think at that stage the scared enough, they've just been given the heart failure and I wouldn't change the name of it because I know sometimes it's arguments around that, heart failure is there for a reason. But if you then say yes, you've got heart failure. I think we need to talk about your CPR. Uh, sort of status and what your feelings are, I just think that that would be too much in a very short space. *Heart Failure Nurse*  Absolutely. I think at any point, maybe not when you're straight diagnosed because it is a lot to take you on obviously. You've been hit with these like you know, heart failure. *Patient*  A good discussion I think is the timing of it. Picking up on cues, whether that be sort of recent admissions, whether there's been a significant deterioration or whether the patient brings it up themselves, we have a number of patients who in conversation happens to say, you know, ‘and don't be resuscitating me love’ and it leads then on to you know that that conversation. *Heart Failure Nurse*  I think sometimes doctors leave those decisions till very late to have them and I think that can be really difficult. Especially patients with heart failure and things like internal cardiac defibrillators and things like that are left to last minute to be switched off, when conversations need to be had much earlier in sort of their disease trajectory really. *Palliative Care Nurse*  As early as possible, from diagnosis really I can understand the situation whereby you're making the diagnosis and breaking the news of the diagnosis to your patient. You don't want to immediately follow it up with end of life, with CPR discussions. From that point onwards the clock is ticking, and if you leave it too late, then the patients going into present peri-arrest or in cardiac arrest without having had a conversation ahead of time and ability to advocate for themselves and express their own wishes. That's the main thing I think about. I see people in ED without these decisions. *Emergency Department Consultant Physician*  I don't routinely have the discussion with everyone when they're first diagnosed with heart failure, because quite often their ejection fractions will improve after it started on the optimal therapy and their risk of sudden cardiac death will be decreasing and actually there's an awful lot for them to be getting the heads around with the new diagnosis of heart failure in any case. I tend not to discuss it with them completely up front. It will be more like, you know, the been to clinic a few times are already on tablets and it's kind of start thinking about it. *Cardiology Registrar Physician* |
|  | Record taking | Documenting the discussions had clearly and if appropriate filling in forms such as DNACPR form | So, the discussion is about priorities of care and values and goals for care and if it's appropriate to, CPR may come into that that discussion. And then, so that becomes about priorities and choices and then, and obviously then the potential to record that conversation in a way that it hopefully informs future care. *General Practitioner*  There was a signed form in the room and still the ambulance staff started CPR... So the good outcome is that the patients voice always gets heard and followed by everybody else which is why documentation in theory should be helpful. It's just in my experience which is I realize that's a one off, it doesn't always get followed. *General Practitioner*  And so we went to reassess them and re-jig their care plans. And I suppose that's the group where as part of that you'd be thinking about priorities of care and then and then, because you're doing that, you think about how do I record this, not only in the practice, but with a out of hours team with potentially with hospital teams and so on and so forth. And then it's the purple forms or the green forms as they are in elsewhere or whatever and it's and the CPR do not resuscitate comes onto those.  *General Practitioner*  Everybody's on the same page and everything's documented the way it should be. *General Practitioner*  I would record my conversation in the medical notes and then make sure I handed that over to somebody senior to make sure then that form is completed. *Palliative Care Nurse*  So I've same RESPECT forms [advance care planning documentation] come out that say things like level 2 and Level 3 care. Well, I know what that means because I work in a hospital setting, predominantly in the Community. Community colleagues, many of them won't have a clue because they have not worked in those environments and certainly patients wouldn't have clue what that means. So language of RESPECT forms I see often change as things are added; ward based care, what does that mean? We have wards in the Community, if a community practitioner read that and the person was in a community ward, would they think not to escalate them to hospital on the basis of that so. *Care of the Elderly Consultant Physician*  Yep, so family being involved in the conversation wherever it occurs, information sharing to improve across provider organizations, one record will be lovely. And coding of information so you know as we complete an advanced care plan, there are coding opportunities around resuscitation and escalation of care. Special notes can be added into system one, so paramedics can see that and other healthcare practitioners. The sharing of information with care homes in particular on discharge from hospital and moving away from paper-based RESPECT forms which just seems so archaic, to electronic forms that can be seen across the system. *Care of the Elderly Consultant Physician*  And no one ever thought on the Thursday or the Friday to maybe have that conversation when they could get the family in and suddenly the nursing team, because they're usually the first on the scene. I just having to start it because it's not documented anywhere, they have to follow the process. *Heart Failure Nurse*  In our team's clinical nurse specialist we actually don't complete the RESPECT forms as such. I think that clinicians responsible for their care do that. But I would add to a respect from that's been completed about other things, if patients added extra things that I felt was important. *Palliative Care Nurse*  And it also to encourage each other also to know each, you know these patients are quite sick, they've got a lot of cardio problems, you know, we're also from a safety point of view perhaps, and it would be mortified if we're resuscitating somebody that had a form. So we do have it quite prominently on our documentation and I just I'm normally go into it and say ohh have you got any preplanned decisions about your care going forward or have you ever had a red resuscitation form. *Heart Failure Nurse* |
| **Who does it?** | Multidisciplinary team | Having different members of the multidisciplinary team present | So we do some heart failure MDTS and we are going to be doing some advanced communication skills because we're aware that we don't have these discussions very well or we've not had training in them. *Cardiology Consultant Physician*  So a lot of what we're doing is backing up their discussions and saying, yeah, we agree, we don't think there was anything, but that's very different from having that conversation with that patient and those relatives and looking after them right at the end of their life. They're at home, they're in the community. I say increasing diuretics. It's the nurses that are doing the end of life care. *Palliative Care Consultant Physician*  But we outreach, so we go out to the other wards because there's heart failure patients across the trust that most of the minds of elderly medicine, but we get surgical and orthopaedics and they don't get the same level of treatment.  *Heart Failure Nurse*  It's all the GP at the moment, and in fairness, a lot of the GPs are very much guided by what we say and they know that we've got that relationship with the patients and many times we get feedback that the conversation was a lot easier because we've actually done a lot of the work for them and had had those conversations. But as it stands at the moment, yeah, it's just GPS and obviously in the task should be centred, it will be the medics. *Heart Failure Nurse*  I think the other thing to say is we often have been in with conversations, but we would stay behind and just explain it and give more information after someone's left as well, because sometimes you can just see the patients just pondering what's happened. What does that mean. So I think it's about just further information given, and listening really as well. *Palliative Care Nurse* |
|  | Training | The training a healthcare professional has undertaken for CPR discussions | I am not convinced I've ever had any formal training around CPR discussions. It's something I do very regularly and obviously we have communication skills at medical school. I don't ever recall having any formal training on discussing cardiopulmonary resuscitation with patients or relatives. *Cardiology Consultant Physician*  So we do some heart failure MDTS and we are going to be doing some advanced communication skills because we're aware that we don't have these discussions very well or we've not had training in them. *Cardiology Consultant Physician*  It it's not really taught to nurses, you know it. It's something that, you know, you might go on your BLS [basic life support] and your ILS [intermediate life support], in CCU [coronary care unit] used to do ALS [advanced life support], but it was only like a little small component where you'd actually talk about the do it should you have CPR on certain patients. It's just something you pick up from interaction with doctors or you could probably argue your experiences of watching CPR done on patients. *Heart Failure Nurse*  No, no, I've not had any advanced communication conversations. I mean, I've done the role for a number of years. And I've watched doctors give the same information or give similar information, but no, there's no formal training I've seen. *Heart Failure Nurse*  Yes. So all myself and all of the team have had palliative sessions and with the local Hospice and the community groups provide, you know, understanding of the palliative sides of things, the advanced care planning. … and all of my team as well have undergone advanced communication skills. *Heart Failure Nurse*  I am, so you know, when I was still in hospital medicine, I did the advanced Life Support courses and things as well. I'm sure it came up during those sorts of discussions and I don't remember ever being part of my clinical training in hospital medicine, but it's a long time ago. *General Practitioner*  So in my, in my limited experience, the people who've been more likely to ask me are advanced nurse practitioners, community matrons and so on and so forth. And what I've always assumed it was something missing from their training. *General Practitioner*  everybody else says ‘I've not been trained’, but that's just a cop out answer to that. What does, tell me what the training would look like then, well, what training do you need? You know, I'm not saying we shouldn't train, I'm just challenging people's notion that there's somehow I go and sit in a seminar room for half an hour and I'll come out of it and I'll suddenly be able to do it. No, you won't. Because it's more complex than that. *General Practitioner*  There's something wrong with the training, whether it’s with nursing, physiotherapy, occupational therapy, pharmacy, or medicine, there's something that is failing patients. *Stroke Nurse*  So you know when the newer forms came out … There was a lot of retraining at that time in terms of which paperwork to use, how to fill out appropriately, who to involve in those discussions. I don't think that there was kind of formal simulated consultation sessions,… but there were certainly seminars and tutorials on how to, you know when to have those conversations and who to have them with, and trying to make everything a little bit more transparent and patient centred than it perhaps was historically so. *General Practitioner*  But most of those have had the benefit of, you know, fairly comprehensive communication skills training throughout undergraduate practice and that probably prepares you for most situations to a degree. And then I think the expectation, at least from my experience, is the rest of it is kind of picked up by watching senior colleagues do it and then doing it with their supervision and their help and then eventually doing it by yourself. *General Practitioner*  I think an element of training around communication for health and social care practicians is essential. I wouldn't necessarily say training around advanced care planning and resuscitation is essential for all health and social care workers. *Care of the Elderly Consultant Physician*  I'm not attended a training course for it. I've had people give me mentorship and individual training and I’ve been involved in training during my training program with teaching session on it. *Emergency Department Consultant Physician*  Obviously, through being part of the cardiac arrest team for probably about 15 years, I've done ALS four times. And so obviously within that scope. And I've also done some additional training with Advanced Communications and DNACPR conversations on the back of my dissertation. And how to approach and start those conversations and feel more confident discussing it. *Heart Failure Nurse*  I think the minimum training is you know, like a basic life support course or an intermediate life support course or an, you know, the advanced one. I think they’re really good for kind of giving you the technical skills of what it is to actually perform CPR and what's required and what it is to be part of the team and. I think that with regards to the kind of communication side of things, that's a bit less. In my experience, a bit less taught in a kind of formal way and I think it's kind of alluded to in ALS courses and it’s spoken about? And but I think that the line I found most useful is through actually being at a cardiac arrest and seeing what other people do, and almost like almost cultural learning space. *Cardiology Registrar Physician*  Advanced Communication Skills Course I've been on and I'm trying to remember. I don't think we got really very much training on it at all in medical school. And so I think it's really all come after that. Obviously the legal side of things has changed a bit since I was in medical school, I'm in terms of, you know, having to inform patients, *etcetera* because before you didn't really have to. And so it didn't happen very much. But yes, I suppose in terms of training, it's mainly been from a communication point of view rather than specifically resuscitation. *Palliative Care Consultant Physician*  So it's the whole, it's the whole process and how I was going to say presentation, how you present. It's not like really, it's how you interact, isn't it, it's how you broach it. And there was talk about training, you know, and all that sort of thing and it's, you know, I think it's very important to talk, to train somebody up if they're going to be involved in that, to train somebody up, to do that properly and give them, shadow people who can do it well, talk to patients and carers. In similar situations, a bit like you're doing here, but you know further down the line. *Patient* |
|  | Communication skills | Can both facilitate and hinder CPR discussions | The best conversations I've had have been when I've been able, so the the best conversations are when I one know my patients and when I know my patients well and the ones that I find the most satisfying are the ones where we can talk about things openly and honestly. *Cardiology Consultant Physician*  I suppose, good communications skills, you know, and that they know the right time, that they can read the patients body language, they know maybe went to back off you know sometimes and you know you the response when you initiate the conversation mightn’t be the best and that maybe it's good that there's someone there with them and as well. That you maybe might need to enrol the help of an interpreter, and that you're empathetic and sympathetic, that you would and use kind of clear, concise language. *Heart Failure Nurse*  Because very often consent is not informed consent and if it’s not informed, it’s not consent. Because Clinicians are really, really poor at communicating.  *Stroke Nurse*  And there's an art to taking them through that, that bit of communication. And this is where training comes in, because not everybody got that. Again, it takes effort, it takes it investment to teach people that art, and it can be taught. Anybody can sell it. Really anybody can sell a lot of healthcare, a lot of rehabilitative specialty involves selling skills because you know you need to find out where the patient is, where they want to be and how much they know where they're coming from. *Stroke Nurse*  And I think as long as conversations are sensitive and they're done with compassion, really you can't go very wrong. I think it's when it's rushed, when somebody comes in and stands at the end of the bed and tries to have it and just say we're not gonna resuscitate you, that it goes very badly. So I think when someone sits down, uses the communication skills that they have, does it in a sensitive way. Often it can go very well. *Palliative Care Nurse*  So I think it's all very individual the circumstances that there are and that's why it does require advanced communication skills to have these conversations and not in isolation. *Palliative Care Consultant Physician*  Yeah. I just think about being open and honest, but also taking a bit of a lead from them as well because it's difficult if somebody is in complete denial. *Carer*  But I think it's trying to keep the conversation open. And not trained to make a judgment, not trying to sort of, people don't really listen do they, they just want to talk don't they and I think you you've got to be mindful of that.  *Heart Failure Nurse*  And then I guess on the other side of it and understanding about the communication skills required and even just to set up a conversation. Which yeah, would be the skills I think people would need. *Palliative Care Consultant Physician* |
|  | Confidence | The preparation, training and experience that one has giving confidence to discuss CPR, either to family, people with heart failure or healthcare professional. | For the patient's point of view, I think it should be or it should be, or you hope it would be somebody who has those skills; confidence, capabilities to do. Because done badly it's awful. *General Practitioner*  It's embarrassment and uncertainty. People are not experienced in these conversations. They don't the confidence.  *Stroke Nurse*  So, I think to think it's one person's role is where we've run into problems with do not resuscitate all along. That anyone can have those conversations as long as they feel comfortable and feel able to do so. *Heart Failure Nurse*  But I do recognize, and I suppose from having worked with a number of colleagues, both medical and allied health professionals over the years, that people find these conversations really difficult and so there's something about the confidence of the practitioner. Some of it comes from experience, I get that. *General Practitioner*  And I think sometimes if a patient said actually if they didn't have a respect from in place and they explicit said to me, I wouldn't want that, then I would feel confident in going forward with that. *Palliative Care Nurse*  [I feel] very confident in having those conversations. Proposing it is an intervention when I think it's appropriate and recommending that we don't resuscitate when I think it would be futile or not in the persons interests. *Care of the Elderly Consultant Physician*  And I think acknowledging that uncertainty with patients is important, but can also be quite anxiety provoking, you know, like I I think if you, if you kind of said to each patient what you thought they're likely prognosis is, firstly you’re probably wrong. But secondly, it's not, it's not going to be kind of positive interaction really for that patient, unless you can say something a bit more concrete and for that you need a trajectory and that's why I kind of say that it's much, it's much easier when you've got someone who has objective signs of ongoing deterioration. You know their renal function is going getting worse…. *Cardiology Registrar Physician*  And I think people find that really difficult to talk about because, you know, we're scientists at the end of the day. And I think having a conversation about what we think might happen in the future is more the art of medicine and seeing the progression of someone over time and especially for non-consultant grade doctors who potentially might meet this patient once you don't have the benefit of a longitudinal view in the same way that an old-fashioned General practitioner might have done. And so I think those things make it really difficult to take the bull by the horns and say I'm going to do this because it's the right thing to do for the patient. *Cardiology Registrar Physician*  And someone who feels that they have enough confidence to have that conversation so they're not as nervous or stumbling as they might be, and I think someone who can, I guess, bring the patient along in terms of recognizing how and well they are or where they're up to with their illness. And so that it's more of a, they arrive at the same position together really. *Palliative Care Consultant Physician* |
|  | Resources | The time, space, leaflets/information available to aide CPR discussions. | this mantra at the moment is that the GP is too busy and it's too hard to get appointment, so getting over those barriers is another challenge. *Heart Failure Nurse*  we must identify that this gentleman is deteriorating early, allow the family to be at the bedside and that the medical team are present to talk through why these stages cannot be undone so that they can see it's not that people have given up, it's just that there's not anything extra to do and so its a big utilization of resources. But for 45 minutes, I think it's worth it for somebody and their family to feel like it wasn't that I gave up. It was just that there was nothing left to do, and we've reached the end of the line. I think that was a difficult one that worked out in the end but make it the middle of the night on a Saturday and that wouldn't have been the case. *Cardiology Registrar Physician*  It can be quite a challenge, obviously. You know, when I started in this job, we have, we're quite fortunate we have 30 minutes with every patient which is luxury compared to some. But by the time you go through a full clinical examination and medication review, answer every other question that they might have, it's very difficult then to be able to dedicate a good proportion of your time, because often you know you, you open that can of worms almost to, you know with that, once you start talking about do not resuscitate it and moves on to what their wishes are for an ACP. And very often they've got their own experiences with that from other family members and it can be, it can be a longer consultation and you've really got time for that. I'd hate to think that comes across like you’re trying to rush people, but sometimes we are limited to the time we've got as well, aren't we? *Heart Failure Nurse*  And half an hour can be spared when it's gonna make a lifelong difference to the people left behind. *General Practitioner*  That you allow them time to give questions, that they're not appeared rushed and that they can come back to you again at a later date if they do have any questions, that they have a contact number for, you know for them to get back on to you. *Heart Failure Nurse*  Time. We just want, you know, we're under so much time pressure. … But these problems were there beforehand [before the pandemic] and they've just been kind of made slightly worse by everything that's gone on in the last few years. *General Practitioner*  And the biggest thing other than the misunderstanding, is time, you know, if you, like in our work, if you want to do a comprehensive geriatric assessment covering all modalities of concerns that the patient has, advance care planning, takes time. … So, you can see why it doesn't happen routinely in primary care. If you've only got between 7 and 10 minutes for an appointment specifically, for what person has concerns about which often isn't their advanced care plan. *Care of the Elderly Consultant Physician*  CPR, do not resuscitate form ultimately is a service need, it's not the patients need so it's whoever in the service has the skills, resources, time and relationship. *General Practitioner*  … we use the booklet what to do, what happens when my heart stops and try to encourage people to read it just to try and process what it means to be resuscitated. *Heart Failure Nurse*  I went to a website which was a compassion in dying. That led me on to something which I assume was a sister organization called ‘My Decisions’, I think, which provides a template, and you fill in, you answer the questions and then it. Then it writes it for you. And produces a view with the PDF. So I had to go at that. Found it rather difficult. *Patient*  … I know there are various different patient information leaflets such as what happens in my heart stops that kind of thing to talk them through the through it. *Palliative Care Consultant Physician*  we use the booklet what to do, what happens when my heart stops and try to encourage people to read it just to try and process what it means to be resuscitated. *Heart Failure Nurse* |
|  | Seniority and specialty | both effect knowledge and understanding of CPR, relationship with patient, resources, experiences and confidence around CPR discussion. | And those sorts of discussions shouldn't be delayed until you can have a cardiologist come and offer their opinion. I think it's that's the responsibility of all of us, really. *Cardiology Registrar Physician*  It's you know your privacy to discuss it and that the that you use you know sort of easy understandable language, that you're not frightening them and that's, you know, and that you're being realistic about what the outcome could be and maybe it's more than it's not just A once off conversation, maybe it's you revisit it and maybe with another health professional maybe the doctor or the consultant that they would give their input to and that they know that they should inform their family of their decision as well. *Heart Failure Nurse*  I think it should be a member of the specialty team, I think, especially as heart failure becomes increasingly complicated as a disease.  *Cardiology Registrar Physician*  I think as long as somebody is suitably qualified, trained and experienced enough to have those conversations, then it's absolutely appropriate. I've seen palliative care nurses have much more thorough and patient centred consultations than consultants who’ve been on the job for 20 years, so I don't think your job title should be what determines that. It should be that you've been appropriately trained and feel confident and comfortable having those conversations. *General Practitioner*  I think obviously the palliative care team are well placed to have these conversations because they can look at a patient from a comorbid perspective, as can geriatricians, as can patients GPs once they've been given the information to understand the cause of their breathlessness and their symptoms. And I think the people who should not be put in the position to have to do it are ITU teams. And ED physicians, it's not fair. *Cardiology Registrar Physician*  In palliative care, I think you've got the ideal setting provided the palliative care doctors made a, and I do think it has to be a doctor, really, has made a good relationship with the patient and the two are coming from the same place. *General Practitioner*  But yeah, it doesn't have to be, you know, it's not got to be the heart failure specialist nurse or got to be the GP or got to be the cardiologist or got to be the palliative care person either. *General Practitioner*  I really do think that it should not just be confined to GPs, it should not just be confined to consultants, should not just be confined to poor junior doctors who are swimming in this great vortex of ‘help, what have I done, what did I, what have I done in the previous life to be put through this shit?’ I think it really should be something that should be going on between staff nurses, junior staff nurses, if healthcare assistants were brilliant, were intelligent enough to have this conversation, they should be doing it as well, dentist, anybody who, pharmacists, anybody who has dealing with a patient who is in a better place to know what's going on with CPR than the patient does, should be able to have this sort of conversation. *Stroke Nurse*  It must be harder for the health care professional who's just seeing you once a year or whatever, and doesn't know you really, although you know read your notes. It must be harder for them and also harder for me as the patient, to bring this topic up or to discuss it in those, whereas with somebody like nurse specialist who I have got to know really quite well over the last 12 months. It's much easier, and yet he's also a professional who knows the knows the routine, knows the situation, knows the, you know what the what the what the decision points might be, and so on, yeah. *Patient*  I don't think a lot of cardiologists are very good at having these decisions, these discussions and that's probably something about the fact that we're quite an active specialty in that there are always things that we can do. And it's my experience that quite often these discussions happen quite late, someone’s 80 and has got cardiogenic shock and they're on inotropes before we think about having those discussions. *Cardiology Consultant Physician*  I think all the medical professionals I think from even the paramedics. Heart failure nurse, your consultants. Maybe even if you've been admitted to hospital and you're maybe under a different consultant or you're in a different hospital than you're used to. I think everybody should be, should be made aware of it. *Patient*  Well, I think anyone can initiate the conversation, but maybe if it's going to be documented, I don't think they take my hearsay, you know, honest and that that it would have to be, and you know the clinician or the consultant which would have to document it. *Heart Failure Nurse*  To an extent, this is gonna be a bit woolly, but I think anyone can commence the discussion around advanced care planning. A family member could, the person themself could initiate it. *Care of the Elderly Consultant Physician*  But, in the context of health care should be any qualified professional who knows the prognosis of the condition that we are talking about and also the available options when it comes to prolonging the life. So in terms of knowing what devices can be used, knowing what drugs can be used, what present care interventions can be used to stave off death. If you've got a decent understanding of that, together with the prognosis of the condition we are talking about any qualified professional can. *Care of the Elderly Consultant Physician*  I personally feel that it should be part of any management with, you know, the medical team, specialist nurses, or even practice nurses or district nurses. Not so much kind of fully discussion up to the form, but actually to be able to, even just sort of plant the seed or start that little ball. *Heart Failure Nurse*  And I feel like it's better for a senior member in the medical team to have the discussion rather than the junior Members because they're more in a place of tending the patients. They have more experience to tell them or this is what the prognosis is or this is how the course of the illness is going to be. So I think like registrar or consultant would be best place to have the discussion. *Junior Doctor*  I think at this moment in time, I would be more confident if I spoke to a cardiologist of some description. *Patient*  My experience of the heart failure team, this isn't a criticism, this is just a reflection of what their job is, is that they deal with heart failure brilliantly, but they don't deal with everything else that's going on. They pass that back to the GP. *General Practitioner* |
| **What should happen** | Rapport, Trust | Building relationship with patients prior to initiating discussions around CPR. | I think that probably starts with having a good relationship with your patient and being able to have an open discussion with them. *Cardiology Consultant Physician*  So, the consequences of, so there's things that can alter the way that you're, relationship with the patients are. So, I've had, I've been involved in some patients where that conversation is completely broken down. The relationship between the medical team and the patients, particularly when there's been opposition to what patients and relatives and staff have thought has been the right thing to do so makes a bad conversation. *Cardiology Consultant Physician*  But again, if you've got a good relationship and a longer-term relationship with a patient, I think often if patients are really sick, it feels it feels like a very final thing to bring the relatives in the obviously, we need to discuss it with relatives. *Cardiology Consultant Physician*  And actually, you start to get a more of a rapport with a patients because we see them more often. *Heart Failure Nurse*  I've known patients for 6, 7, 8 years. I still see them every so often dotting around the hospital for other reasons and it's always a hello. That comes with other problems because there is uh at the end of there is a trajectory for heart failure, which can also be quite sad because you've developed that relationship. *Heart Failure Nurse*  Am I think from just personal and professional experience working on the wards and seeing CPR in an acute setting. And having conversations and you know, that relationship with patients in the Community who are sort of a more of a chronic stage of their illness and having decisions and discussions around what they want. *Heart Failure Nurse*  I hesitate on relationship because it used in so many different ways and I think you can do that on a first conversation, you know, I’m starting to contradict to myself now, you can establish a relationship on a first conversation, I'm not saying relationship is the same thing as you've got to have known them since they were a child. *General Practitioner*  Their cardiologists have known them for a long time. But often we get referrals when they've made the decision to sort of change the focus of care and then want us to come and have a decision, a discussion about advanced care planning. When actually the people that have known them the best are in a better position to do that because they've already got rapport. *Palliative Care Nurse*  So, I think it's been easier and I think I've definitely seen how, particularly these rooms as well, one to one care that, you can quickly build a rapport with a relative. That's the lovely thing about here is that the relative is here, the wife of our daughter. It's more protected and supported. But also they feel. You know the rapports there, isn't it? *Heart Failure Nurse*  Because you know, in an ideal world and for the last couple of years been very far from that. In ideal world the GP is somebody who actually does know you. Umm. And this particular person I had seen him, you know, with various, you know I've had quite a lot of health care needs over the years, so I have seen him quite a lot and I felt that it was somebody. This particular person in the practice was somebody that I got on very well with who did know me. So I would have wanted him yes to be, I expected him to be a helpful advisor. *Patient*  I was absolutely fine that the people to whom I have entrusted, my father's care were leading that conversation. *Carer*  Well, an ideal situation would be somebody who knows the patients at least once before, obviously that might not always be possible, but someone who has a bit of a relationship with them so that they're not coming at it cold. *Palliative Care Consultant Physician*  I think like not sugar coating anything. I’m one of these people that like to know that the nitty gritty and like to know exactly. So I think just if it's a discussion about it, tell the people exactly where it is, what it does and how using it can be beneficial to the person that needs it. *Patient*  I've entrusted these people to act in the best interests of my father. And I would hope that they are going to take the correct decision at the time, which is based on not just the purely the medical outcome that it's based on the quality of life, type of decision. Umm. And they brought it up and I would imagine that in that situation in in time they would always bring it up. That would be my expectation. Uh, so if they hadn't had brought it up, I wouldn't be turning around saying so. Hey, you know, just wanna let you know, guys, if he has a heart attack, don't bring him around again. You know, if he has anything like that or another stroke, don't bring him around again. It's odd, I just kind of assumed that that's what they do or not do. *Carer*  I think that you can colour the whole of the rest of the admission and actually and mean that people have very negative associations with that healthcare episode, which could be really bad for people with heart failure because they need to kind of have faith you know it's a chronic illness. They will be having lots of contact with healthcare professionals and you don't want to kind of ruin that relationship by saying something really insensitive or inappropriate. *Cardiology Registrar Physician*  It that's the hardest thing I find, when you don't know someone very well, is is to having that conversation where it needs something and it's not just a random person you've met and think well, nobody's ever said that to me before that must mean I'm either dying or she just, you know, that's her thing, you know?  *Heart Failure Nurse* |
|  | Multiple discussions, advanced care plan | Having a CPR discussion as part of treatment plan, over multiple clinical interactions, focussing on holistic patient care. | I think exploring a patient's ideas, concerns and expectations around their health. Is the opening to these conversations. *Cardiology Registrar Physician*  So yeah, I'm up to date with obviously the use of RESPECT forms, which is what we use as our advanced care planning tool and see cardiopulmonary resuscitation discussions simply as a part of that advanced care planning. *Care of the Elderly Consultant Physician*  Yeah, I think so. I think it's just about the skills that you need to have and I think whenever I'm trying to have those conversations, I try not to have them in isolation. I think that is more difficult in a in acute settings. But, yeah, it's, for me it's about not doing them in isolation and actually making sure the within context of part of another conversation. *Palliative Care Consultant Physician*  It's not just a once off conversation, maybe it's you revisit it and maybe with another health professional maybe the doctor or the consultant that they would give their input to and that they know that they should inform their family of their decision as well. *Heart Failure Nurse*  You can have very good conversations towards the ends of patients’ lives as well. Yeah, if they understand exactly what's happening. If there's been some discussion about it before, if it's not something the patients have thought about, if it's something the patients have thought about for a long period of time, that often is helpful or if other healthcare professionals have brought it up as something for the patients to talk about in advance, that's usually what's helpful, I think. *Cardiology Consultant Physician*  I've got a patient at the moment who is, clearly getting worse, and I can see him getting worse. He's not interested at all in having those discussions with me. He wants us to investigate everything with him, even though he's much too old to be thinking about advanced heart failure therapy. We're having to talk about those kind of things with him so that he feels like we've done everything. But that's a barrier. And yeah, it doesn't mean you shouldn't have that conversation or initiate that conversation. But it's also important that you know when to stop having that conversation and do it very gently and over a period of time because it, I don't think it has to be a single conversation all of the time. I think it can absolutely be a process. And a single conversation works really well for some patients and for other patients. It very much is a process. *Cardiology Consultant Physician*  So I had a series of conversations with this guy about priorities for care, and we agreed that CPR was not unlikely to be successful for him or helpful to him. *General Practitioner*  it's about putting something into place and following it up and potentially revising it and so on. *General Practitioner*  So, you know this community matron have been seeing this person for weeks and months and then suddenly the GP gets rolled out to go and do the form. And I'm just like, that is just so inappropriate. *General Practitioner*  I think it took 3 maybe four fairly lengthy home visits before he sort of accepted that having a DNACPR form was appropriate. And so that's a that's a big investment of time in one patient and it's appropriate to do it because it needs to be done. … So for the patient is probably quite a good thing because he had the opportunity to think things through, have these repeated discussions and then come to that agreement with us over to time. But it would have been nice to do it in maybe one or two visits instead. And being selfish, well not selfish, but thinking about the resource allocation of that time and money that's spent having that one conversation four times and going around in circles. *General Practitioner*  I think you’ve still got to broach the subject, but it's like for me it's like that gentle prodding. So it's not something that could be necessarily done in one sitting. I think it's you introduce the topic. If you've got the time, of course not. Not everybody's got the time. I mean, as in, you know, it's not imminent that is gonna die. But I think if you've got somebody on treatment, I think that conversation needs to keep happening like a slow drip feed really. *Carer*  So that's what I mean by a drip feed that could that seed could be sewn quite early on by thinking, gosh, let me find of the year. You know, my chance of survival is 50% in five years. All that's OK. You could go up 50% means that I could stay for another 10. Maybe, you know, but at least it's. You've thought it through. And then if treatment starts not to work, you can move those statistics down. So, somebody gradually gets to understand. *Carer*  Not so much kind of fully discussion up to the form, but actually to be able to, even just sort of plant the seed or or start that little ball. You know, I feel sometimes when we do that in clinics quite a bit is we just try to gently move into to that territory. *Heart Failure Nurse*  And I'm impressed also, by the way, he keeps come back to it, not not in a pushy way at all. And the reason I haven't is simply because I've been rather busy. I'm back at work. I work for myself. Editing books? Not medical ones these days a different kind. So I've been just too busy to think about it. It's my excuse. It's not a very good excuse, but yeah. And I suppose there's an element of not quite wanting to think about it. I don't. I'm not sure because I do want to think about it these consciously, I want to think about it, but it's still quite difficult to make yourself do it. *Patient*  he doesn't bring it up a lot. But you know, from time to time as mention… He did it very at a very good time, the minute time mentioned it, I mean, he was in into my room and the ward saying let's talk about this outcome back when I’ve got some time, you know. And and the plan is to deal with the emergency healthcare plan and then move on to the advance directive. And the ball’s in my court and I need to get on with it. *Patient*  So definitely kind of we revisit those kind of discussions then and it might be the decisions to be made that someone would be for a resuscitation, but then events transpire and actually things are much more complicated and the chances of a successful outcome are much lower. And then that would be a time that you obviously have to reevaluate it. *Cardiology Registrar Physician* |
|  | Patient involvement | Addressing the person with heart failure’s ideas, concerns and expectations | Having patients who are engaged with that conversation almost before we start having it makes a really good conversation. *Cardiology Consultant Physician*  Where both you and the patient have some idea that that might be something that we're going to talk about and it doesn't come as a complete surprise to the patient. *Cardiology Consultant Physician*  You should have an idea of what their wishes would be before you start that conversation. And I've seen some consultants and I think I mentioned in our initial email his start point was ‘so where do you think we are? Going forward, you what do you think we should be doing going forward?’ And then he brought in the conversation. It wasn't just a hello, Mr. Smith. I think we should do this now, OK. *Heart Failure Nurse*  A good discussion I think is the timing of it. Picking up on cues, whether that be sort of recent admissions, whether there's been a significant deterioration or whether the patient brings it up themselves, we have a number of patients who in conversation happens to say, you know, ‘and don't be resuscitating me love’ and it leads then on to you know that that conversation. *Heart Failure Nurse*  I think it's it wherever is the best for the patients and I think you've got to be led by them. You know if it was a busy clinic and they opened up the conversation, I probably wouldn't shut it down. But I probably wouldn't think this was necessarily the best place to explore all the other options and in those situations will probably do what we needed to do at that point and come back and revisit it really. *Heart Failure Nurse*  And you know, what is the person's own choice, personal choice. Do they do they want to be resuscitated and have they made an informed decision? *Heart Failure Nurse*  It was more that my dad had had the conversations with all of us. And he'd expressed his wish. And when I was relaying back to the various members of the family that I've been having these conversations with or been asked the questions by the care home and the nursing home. What will the rest of the family members were coming back saying, well, yes, we agree. We've had those conversations ourselves and that so. That they kind of happened. *Carer*  And it can often be a really quick discussion. Where you just say if you ever thought about this before and people will then say yes I have and it's absolutely not something I would want and they’ve clearly thought about it carefully beforehand. *Cardiology Registrar Physician*  I think a good discussion would be where, it's sort of of 2 way discussion or where you’re involving the patient, their the family. Where the patient wants the family to be involved and you know, sort of taking into account like what the wider team thinks as well. And sort of making sure that a common ground is reached, like that's what the patient wants and they understand why. *Junior Doctor* |
|  | Family involvement | inviting a person with heart failure to have family present during these conversations | Ideally with some support for some relatives, although sometimes that's not actually helpful because occasionally patients feel a little bit more free, I think to talk about dying without relatives there, at some point you have been relatives into the conversation. But I do find that sometimes patients are a bit more free to admitting their fears and concerns without any relatives there, they almost want to keep going for their relatives and don't wanna think about talking about that because they feel like it's failure. *Cardiology Consultant Physician*  So, in the hospital setting as an inpatient service, I think that should always happen because that's a big decision and it's not just the patient. Uh, and I don't know how, I've heard of patient say oh no, I I want it. And I've heard of patients where the family think he shouldn't have CPR. And you get these dynamics. So, I think you have to be open about that sort of conversation. *Heart Failure Nurse* |
|  | Asking versus telling | Consider whether asking a person their wishes on CPR if it is not an appropriate intervention to offer. | I think there's also an awful lot about the way the question is phrased and how you bring it up. So talking about resuscitation as though it is a good treatment and an option for patients actually is often where you were on the way of dealing with things and sometimes framing it as a medical decision for a treatment that you think is futile for a patient that is dying is a better way of bringing that up. *Cardiology Consultant Physician*  So, the discussion is about priorities of care and values and goals for care and if it's appropriate to, CPR may come into that that discussion. And then, so that becomes about priorities and choices and then, and obviously then the potential to record that conversation in a way that it hopefully informs future care. *General Practitioner*  And so, it's about exploring the patients priorities, concerns, interests. It's about explaining if and how various interventions, including CPR, might support that. And it's about putting something into place and following it up and potentially revising it and so on. So, so good generalist skills will absolutely be the core of that. *General Practitioner*  The commonest trigger, in my experience, is the patient or family. *General Practitioner*  I think that is a decision that should be, that the patient should have a command over. Patients should decide who they want to be involved in that conversation. Because there is the potential for adverse influence. You know, I think it's really, it's gotta be the patients’ decision. So and once you've had that conversation with the patient, to then be able to say to them now, you’ve got your husband, you’ve got your wife, you’ve got your father and your mother, do you wanna talk to them about this or do you want support to talk to them? Or do you want me to take over that part of the conversation and tell them myself? And give them the choice. *Stroke Nurse*  Yeah, it's very hard to visualize it totally. But I think you, if I was well enough and conscious as it were, I would want a discussion rather than a fait accompli. So, you've got a choice. *Patient*  so not having at all, you're robbing somebody other opportunity to be able to express their own wishes, because by the time they need, it's often too late. *Emergency Department Consultant Physician* |
|  | False optimism | Creating a truthful and realistic expectations for the person with heart failure | I do think there is a lot to be said for what happens on the TV in the media and television and, you know, two shocks and you're fine. It's not the brutal, almost right always. I mean, apart from outside of coronary care, that of course adds another complexity to heart failure patients because they do get arrythmias from which you could potentially resuscitate somebody if you if you get to them early enough. *Cardiology Consultant Physician*  is CPR the symptom of a bigger problem that we need to be talking to people about what is the point, the purpose of healthcare, what we need to renegotiate expectations with individual patients but also with society about what the health service is there to do and not to do. *General Practitioner*  I think there's still just that perception that it's more successful than it is. *General Practitioner*  The danger is obviously that you may not be realistic about things, and you can imagine a situation where I might disagree and everybody else is saying ohh no, no, you’re too, you know, you need to go, you know. *Patient*  I think people seem to have false perceptions from TV and film, of what we can actually be achieved and what it's for. *Emergency Department Consultant Physician*  And sometimes people think that a shock is, you know, it's that media thing, isn't it? Where I'll be getting a shock and sitting up with a cup of tea and and then go home and all be pretty well. And it's really trying to break that bubble, burst it, and I do, I’m quite brutal. *Heart failure nurse* |
|  | Common ground | Making a joint decision, agreed by all parties. | I called it, I mean it's you've got to insist on shared understanding before you can reach decisions and you don't do it with a form in front of you. You do it through proper trust building and much of the way that CPR discussions take place alas is the complete negation of that. And we've got to find a way to make it an affirmation of that. And, based on a real understanding which is built on true acceptance of the patient’s wishes and true information that is given in a way that doesn't distress or in any way nudge the patient towards a decision. That would be my summary of what's needs to be done. How to do it is another matter, for you to solve. *General Practitioner* |
| **Experiences** | consequences | Each conversation can cause serious and far-reaching consequences to all involved. | And no one ever thought on the Thursday or the Friday to maybe have that conversation when they could get the family in and suddenly the nursing team, because they're usually the first on the scene. I just having to start it because it's not documented anywhere, they have to follow the process. *Heart Failure Nurse*  I think I think a bad conversation sort of shuts down the whole conversation and it also affects things moving from always as well. And I've had a lot of patients who have not had, as I say that open conversation and have just come home with purple forms. And when we broached the subject and they knew nothing about it, and they become very defensive and I think it does make it difficult to have that conversation in the future then. And I think it's also about what the information that you communicate in that initial conversation. And you know I think we need to ensure that patients know that we're not resuscitating and taking it doesn't mean we're not treating with antibiotics doesn't mean we're not giving you IV furosemide *etcetera, etcetera*. I think people often feel that you know that we have just washed their hands of them and then that's it. So I think it's, it's the contents of what's said and the timing of what said that that is crucial in a lot of this because without that. It just leads to and I think problems in the future when you do wanna open up that that line of communication again. *Heart Failure Nurse*  I guess the consequences, having seen it in coronary care or you know, dealt with the after-effects, shall we say and is they’re upset. Sometimes there's a lot more conflict red forms that have been initiated, and I think it's been initiated by the medical team that the patient doesn't agree with. Because like I guess the person, some of it's about their understanding. What resuscitation offers them at that particular point in their life. There can be sometimes resentment off that feeling that people are giving up on you or that they've somehow not giving you something that they should be giving you, that feeling of, Oh well, I don't like this and I'm going to talk to my daughter about this and… *Heart Failure Nurse* |
|  | emotions | can prevent both clinicians and carers from having discussions around CPR due to fear, guilt and not wanting to upset anyone. | So, although it was quite emotional from, I've known this lady for a long time. We've managed her for a number of years. So, this was obvious she had come to the end of the line with all of this. So, it was quite emotional for me and her, and her family as well. So, well though, see. Well, it was tough, but I think the outcome of you know, it was really positive in that. Fortunately, the GP did go out and everything was put in place, and she did have a very peaceful death at home, supported by everyone. *Heart Failure Nurse*  But they just didn't feel comfortable about the Do not resuscitate. I suppose it's like everybody feels it's a withdrawal of care, doesn't it? … in my experience, we're the ones who fear that people are gonna say that it's not the patients who say it. They're actually relieved when we stop their medication or remove this idea that somebody's gonna bounce on their chest or whatever. And I shouldn't generalize in any way. But yeah, I just think sometimes we’re the people who are fearful, not the patients. *General Practitioner*  And this person where you think you're negotiating this space where you're gonna make, work together to make the daily living better. And then suddenly they'll say, ‘but don't give up on me, I'm frightened of dying’ or whatever It is that basic fear comes in. My God, what have I done wrong in this conversation so far? And then you think no, I haven't done anything with this conversation. It's just that it's still there. And yeah, you just have to deal with that conversation when it comes. *General Practitioner*  And I think you have to be very sensitive and compassionate and understand that it can be a very upsetting and distressing conversation, but equally it can be a very calm and considered conversation where actually that person already knows that they wouldn't want that procedure to happen. *Palliative Care Nurse*  I think sometimes there's a fear that you there's almost like a finality to it. Like, I'm sorry there’s nothing else I can do, we need to talk about CPR now, and that's not necessarily the case, but it does make you push it that little bit further on down the line until you really have to do it and that's possibly not a failing, I think that's a human thing because you don't wanna give people bad news. But at the same time, because you've built up sort of four years of a relationship to then suddenly say this is it. I think the impact is more because they trust you. *Heart Failure Nurse*  I found it difficult having that conversation because I didn't want to go there. But I ended up having to go there. *Carer* |
|  | Culture | can facilitate and be a barrier to CPR discussions through its effect on understanding of CPR, beliefs around death, relationships with family and clinicians. | Yeah. And I think it cardiology, everyone wants to, there's a real reluctance to give up in cardiology and I've seen it in lots and lots of articles because there's always that one more thing and we might get another drug. *HF Nurse*  I don't have direct experience of an outright discussion of that in the modern sense of having the form in front and all that. I've got various experiences over the years of family member involvement. Ethically, the patient herself should be the decider and the family have an ambivalent role around that and it becomes very difficult once you move into other cultures. My experience of Muslim patients and I had very, very large number in my practice is that they don't come out openly in this sphere that there's of course an unfortunate male dominance in in decision making, which one can't do very much about in circumstances like this, and there's perhaps more helpful feeling that life does have a beginning and an end and the Allah will be merciful and so forth. And whatever one's personal beliefs, I think a helpful attitude in in many ways. *General Practitioner*  Yeah, yeah, I suppose one of the things that crossed my mind is that within the broader conversation that are cultural sensitivities that I don't know enough about. And I don't know if it is possible to access those opinions. I mean, you know, there are Muslims, there are Sikhs and all sorts of people within the medical profession who will be able to cover those points. I wouldn't dream of anticipating what they're gonna say. *Stroke Nurse*  I suppose that's where the challenge is, and I think you have to respect people's opinion and it's based on something. It's based on sometimes religion or not to interfere…. And sometimes people think that a shock is, you know, it's that media thing, isn't it? Where? I'll be getting a shock and sitting up with a cup of tea and then go home and all be pretty well. And it's really trying to break that bubble, burst it, and I do, I’m quite brutal. This is not real life, and all the things that are in the booklet. *Heart Failure Nurse*  And that's the difficult thing. And often, you know culturally as well and, you know, local farmers or whoever it might be, you know, like to kind of tell you that everything's all fine when it clearly isn't, and the quality of life, is really poor and. I think it's really drilling the quality of life that's. That's why we get more information from family and that's really helpful. *Heart Failure Nurse*  I think. And certain cultures and religions, find it a particularly difficult conversation to have. The idea that you can't give up. And if those beliefs are very strongly held. *Cardiology Registrar Physician* |
| **THEME 2**  **UNDERSTANDING** | Healthcare professionals’, people with heart failure and their families understanding around disease burden and the process of CPR can aide informed decision making and prevent potential iatrogenic harm. | | |
| **Person’s health status** | Burden of heart failure and comorbidities | The symptom burden that heart failure can cause as well as the physiological processes in heart failure occurring without significant symptom progression e.g. kidney disease, hypotension... | Yeah, he knew. I mean the gentleman that lived with a very poor heart function with a really high symptom burden, you know, fluid overloaded could do very little. But he was alive and his family were around him and that was that was his, you know, I think at one point he was even offered a device and, you know, and he snapped their hands off, you know, like, you know, because it was that extra. …You know, he wanted to stay as long as he could, and we tried to give him that extra chance. *Heart Failure Nurse*  But the reality of it, when these patients at home is maybe a different, different picture and I think it's also the fact that we've got so many comorbidities going on here that if we purely looked at a lot of our patients from a heart failure perspective, we'd actually think they were doing OK. But when we do step back a minute and think take into account the renal dysfunction, the COPD, the dementia, that start and you know it, DNACPR I think is not just one condition is it, it's everything and sometimes it's specialists and certain fields we fail to take all of that on board I think. *Heart Failure Nurse*  I think it's been the definite learning curve for me in terms of, you know, intravenous furosemide and isn't just a quick fix really the there's obviously behind that signs that things are going wrong and people on the downward should actually definitely got my head round that a bit more. And the fact that the mortality after intravenous diuretics is quite high. …. *Heart Failure Nurse*  …the symptoms, were none at the beginning, but they're just slowly, I said slowly and unsteadily, is what I say got worse over the years. *Patient*  I would really hope that patients with heart failure are aware that it's a life limiting diagnosis and whilst I know there are new things being done at all the time and new devices and things that can help prolong, prolong life*, etcetera.* And it's still will probably be their cause of death in the end and shorten their life. *Palliative Care Consultant Physician* |
|  | Perception of patients health | Patient, carer or family member and healthcare professionals’ idea of patients’ health status impacts how well a CPR discussion is received and if no consensus can cause upset. If patients don’t feel they are ‘unwell’ enough to be having a conversation about CPR they are unlikely to engage. | I've had some really good processes with patients where we've talked about CPR as part of those things. I'm thinking in particular about some patients with heart failure, where everything has just gone right, right from the beginning being honest about what the prognosis was to patients wanting to have those kind of discussions and communication in them clearly wanting to understand a bit about what the future, having good education about the syndrome by our heart failure nurses, so that when that discussion happened, they understood that there was everything that we could do that had been done and that CPR was a futile treatment and that in this case stopping the end that you know patients heart stopping was an end of life. *Cardiology Consultant Physician*  A lot of the time, they [clinicians] don't spend enough time trying to understand the patient’s level of knowledge retention and how much they can understand and follow what's being explained to them. *Stroke Nurse*  I think conversations always go badly if you haven't done your homework. … If you don't know the ins and outs of all of these things, you can guarantee a family member or a patient will trip you up and say, well, how can we have this conversation if you don't know everything about me or my relative? *Cardiology Registrar Physician*  I think symptom burden plays a real part in where the patients going to accept it a little bit better? I've got a patient now who, for want the better word, I love dearly. I've known them for five years and they are they’re at sort of transplant type destination at the moment. But their symptom burden is minimal and they can't get the head around that because they're still doing everything they want to do. They're still working full time and to have a conversation about CPR to them is really difficult. *Heart Failure Nurse* |
|  | Prognosis / trajectory /Trigger | Can be difficult to determine in heart failure, especially if patients have been given false optimism. Clinical signs and symptoms or specific situations indicating a deterioration in heart failure prompting healthcare professionals to discuss CPR. | And I think acknowledging that uncertainty with patients is important, but can also be quite anxiety provoking, you know, like I I think if you, if you kind of said to each patient what you thought they're likely prognosis is, firstly you’re probably wrong. But secondly it's not, it's not going to be kind of positive interaction really for that patient, unless you can say something a bit more concrete and for that you need a trajectory and that's why I kind of say that it's much, it's much easier when you've got someone who has objective signs of ongoing deterioration. *Cardiology Registrar Physician*  But their symptom burden is minimal and they can't get the head around that because they're still doing everything they want to do. They're still working full time and to have a conversation about CPR to them is really difficult. *Heart Failure Nurse*  I can think of one patient with heart failure in particular who, he was struggling to accept his worsening prognosis and to have those conversations around CPR and things in the community. And when he was going back to his clinic because he was still motivated. And I think that was rubbing off on his consultant, and they were both trying to think of ways to make him better. Because the patient wasn't accepting it. *General Practitioner*  Yeah, you might have heart failure, but it's still you can still live another 20-30 years. And your heart is failing, and that's kind of what it is. *Carer*  I think acknowledging that uncertainty with patients is important, but can also be quite anxiety provoking, you know, like I I think if you, if you kind of said to each patient what you thought they're likely prognosis is, firstly you’re probably wrong. But secondly it's it's not, it's not going to be kind of positive interaction really for that patient, unless you can say something a bit more concrete and for that you need a trajectory and that's why I kind of say that it's much, it's much easier when you've got someone who has objective signs of ongoing deterioration. *Cardiology Registrar Physician* |
|  | Death is a natural process | Society doesn’t like to talk about death. | So, you're not necessarily saying we're not gonna do this as a medical team and because that always does feel quite negative or as it's a lot, feels a lot more inclusive if the patient comes to the realization that they wouldn't want that anyway. And I'm seeing it as part of a natural process of dying, rather than needing to medicalize it. *Palliative Care Consultant Physician*  we need to be talking to people about what is the point, the purpose of healthcare, what we need to renegotiate expectations with individual patients but also with society about what the health service is there to do and not to do. *GP academic*  In an ideal world the general population would be informed enough to have open discussions as a society about what death look like what does looks like and how we can go about planning to have the death we would want. *Emergency Department consultant physician*  Yeah. And I think there's a certain degree of reluctance then on both sides, because no one really wants to talk about something that's gonna upset someone. *Cardiology Registrar Physician*  I got the impression a bit that other people want didn't want to think about it. Particularly, my sister actually, really didn't want to discuss it or think about it. *Patient* |
|  | Devices | Cardiac devices can be a trigger for conversations, but need specialist knowledge. | Lots of my patients have defibrillators in and early on, if they've got advanced heart failure. I will start talking to them about how they feel about their defibrillator. Do they want their defibrillator to still be active and often that can segway into a conversation about resuscitation. So obviously if the patients bring it up, if the patients bring it up, if they are talking about quality of life. So if it's those kind of discussions that the patients are wanting to have that sort of clinic based. *Cardiology Consultant Physician* |
| **Understand-ing of CPR** | CPR purpose | What CPR is intended to achieve needs to be well and explained | And so, I think in terms of communication skills, really you need, you need to understand the process of CPR to be able to explain that to the patient of what it involves. You need to understand this sort of the aetiology of heart failure, really, and why that's not gonna be effective or whether it would be futile. So that the persons able to understand that as well because I think they may have questions that you may need to answer, and you need to be able to do that confidently. *Palliative Care Nurse* |
|  | CPR outcomes | The possible and most likely outcomes of CPR in a person needs to be understood and clearly explained to patients and family. | That depends on the patient, I think. I think CPR is a really good treatment for people who die too soon or who have an arrhythmia which might cause them to die too soon. My personal opinion is that this is not a very good way for patients to experience their death.  *Cardiology Consultant Physician*  Well for me, … and you just seen it on TV and my understanding was they basically just pump your chest and to try and obviously get your heart going again and get you breathing. And but I didn't realize like how intense it actually is. I mean, they break, you can break your ribs, they can. You know, I was badly bruised. Luckily, I didn't suffer any broken ribs. But the bruising was horrendous. And the pain and it actually gives you a wake-up call, you know. …And I realize now like exactly what it means. *Patient*  I think a bad outcome would be not be able to get the person back. I think that would be really that would be horrendous I think. But obviously I good outcome is getting the person back as I say, like if they've got broken ribs or they're bruised, whatever. But that you have got them back. That's the main thing. You know that can heal. *Patient*  So, a good outcome would be the patient being completely the same way as they were prior to the events. And I think a bad outcome … would be not being in any cognitive or functional capacity as you as you were before. And I think that's very subjective and because what is sort of accepted by one person is not from by someone else. *Heart Failure Nurse*  And I've seen the worst, which is prolonged resuscitation on inappropriate frail patients that with multiple comorbidities. With everything stacked against them and no, you know discernible outcome. Or they come round, and they have no quality of life, no memory. Lost their independence at, lost their dignity, and then they die before they leave hospital anyway, so that is the worst-case scenario. *Heart Failure Nurse* |
|  | Media | Media is a barrier to understanding CPR success, portraying rapid recovery, good success rates and poor technique. It could be used to increase understanding of CPR outcomes | I do think there is a lot to be said for what happens on the TV in the media and television and, you know, two shocks and you're fine. It's not the brutal, almost right always. I mean, apart from outside of coronary care, that of course adds another complexity to heart failure patients because they do get arrythmias from which you could potentially resuscitate somebody if you if you get to them early enough. *Cardiology Consultant Physician*  I suppose that's where the challenge is, and I think you have to respect people's opinion and it's based on something. It's based on sometimes religion or not to interfere…. And sometimes people think that a shock is, you know, it's that media thing, isn't it? Where? I'll be getting a shock and sitting up with a cup of tea and then go home and all be pretty well. And it's really trying to break that bubble, burst it, and I do, I’m quite brutal. This is not real life, and all the things that are in the booklet. *Heart Failure Nurse*  And just general communication, you know the media sound bites around the importance of advanced care planning are really important comms from the CCG's and from local authority as well. *Care of the Elderly Consultant Physician*  So, I suppose from a professional point of view, I guess the resource the Resus Council has a lot of resources and both in terms of the practical and process side of it, but also in terms of trying to decide whether resuscitation is appropriate or not. *Palliative Care Consultant Physician*  It's very difficult and I think it can be used for good. It's generally, I think not generally, perhaps not often used in that way because everybody likes a good Horror Story that they have about when things have gone wrong. It sells papers, unfortunately, but there has been some really decent mainstream publications around the importance of advanced care planning and I think more of that. And I think some of the Richmond Charities and some of the bigger charities and the Hospice movement, you know that are aften very clear around the importance of advanced care planning. It's just whether or not we can get that into the mainstream. *Care of the Elderly Consultant Physician*  I think some of the main issues is what they see on Holby City is very different to what happens in real life and so you don't want their information to be coming from the BBC. *Palliative Care Consultant Physician* |
|  | Training | Training of physical CPR and specific communication skills for CPR discussions for all. | See above |
